# CLEC5A is critical in *Pseudomonas aeruginosa*–induced NET formation and acute lung injury

**DOI:** 10.1172/jci.insight.156613

**Published:** 2022-09-22

**Authors:** Pei-Shan Sung, Yu-Chun Peng, Shao-Ping Yang, Cheng-Hsun Chiu, Shie-Liang Hsieh

**Affiliations:** 1 Genomics Research Center, Academia Sinica, Taipei, Taiwan.; 2 Department of Pediatrics, Chang Gung Children’s Hospital, Taoyuan, Taiwan.; 3 Institute of Clinical Medicine, National Yang Ming Chiao Tung University School of Medicine, Taipei, Taiwan.; 4 Department of Medical Research, Taipei Veterans General Hospital, Taipei, Taiwan.; 5 Institute of Immunology, College of Medicine, National Taiwan University, Taipei, Taiwan.

**Keywords:** Infectious disease, Inflammation, Bacterial infections

## Abstract

*Pseudomonas aeruginosa* is one of the most common nosocomial infections worldwide, and it frequently causes ventilator-associated acute pneumonia in immunocompromised patients. Abundant neutrophil extracellular traps (NETs) contribute to acute lung injury, thereby aggravating ventilator-induced lung damage. While pattern recognition receptors (PRRs) TLR4 and TLR5 are required for host defense against *P*. *aeruginosa* invasion, the PRR responsible for *P*. *aeruginosa*–induced NET formation, proinflammatory cytokine release, and acute lung injury remains unclear. We found that myeloid C-type lectin domain family 5 member A (CLEC5A) interacts with LPS of *P*. *aeruginosa* and is responsible for *P*. *aeruginosa*–induced NET formation and lung inflammation. *P*. *aeruginosa* activates CLEC5A to induce caspase-1–dependent NET formation, but it neither causes gasdermin D (GSDMD) cleavage nor contributes to *P*. *aeruginosa*–induced neutrophil death. Blockade of CLEC5A attenuates *P*. *aeruginosa*–induced NETosis and lung injury, and simultaneous administration of anti-CLEC5A mAb with ciprofloxacin increases survival rate and decreases collagen deposition in the lungs of mice challenged with a lethal dose of *P*. *aeruginosa*. Thus, CLEC5A is a promising therapeutic target to reduce ventilator-associated lung injury and fibrosis in *P*. *aeruginosa*–induced pneumonia.

## Graphical abstract

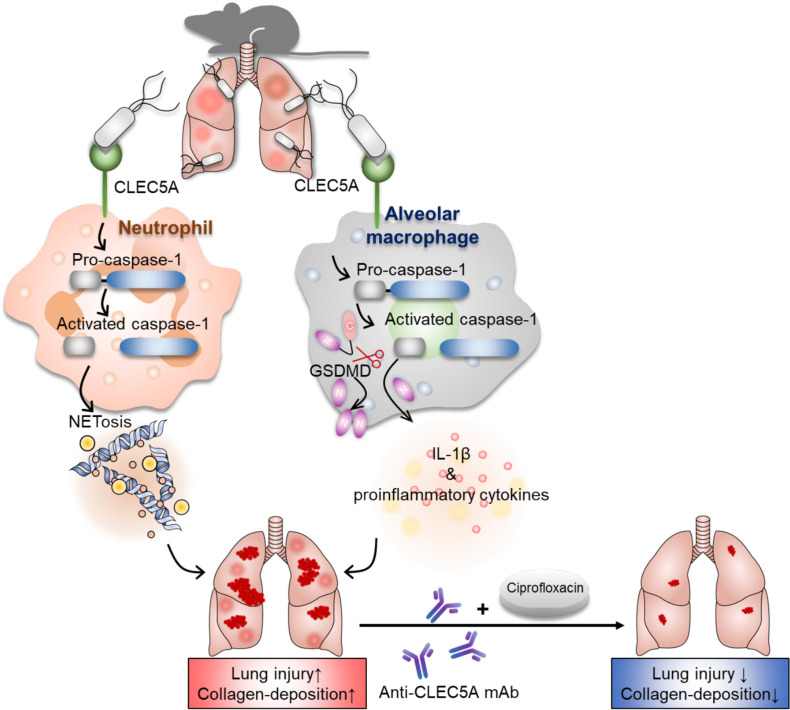

## Introduction

Due to multiple-drug resistance, the modulation of host immunity to complement and enhance antibiotic-mediated cytotoxic effects and reduce tissue damage in bacterial infections has become an urgent need. *Pseudomonas* *aeruginosa*, a Gram-negative, aerobic rod bacterium, is one of the most frequent causes of nosocomial infections following burn injury, radiotherapy, and chemotherapy. In addition, *P*. *aeruginosa* is one of the most common pathogens in ventilator-associated pneumonia and often causes severe lung epithelium damage leading to acute respiratory distress syndrome (ARDS). Therefore, it is crucial to understand the molecular mechanism of *P*. *aeruginosa*–induced lung injury in order to develop better strategies to prevent ARDS and reduce mortality.

Excessive recruitment and activation of neutrophils have been implicated in acute lung injury (ALI) in several disease conditions, and neutrophil-dominant infiltration is frequently associated with acute alveolar damage and intraalveolar hemorrhage (1–3). *P*. *aeruginosa* secretes various toxic exoproducts that damage the epithelium, thereby inducing local inflammation and neutrophil recruitment (4, 5). While neutrophils kill *P*. *aeruginosa* via phagocytosis, they also produce reactive oxygen species (ROS) and release cytotoxic substances, thus provoking lung injury (6). In addition, *P*. *aeruginosa* has been shown to induce neutrophil extracellular trap (NET) formation in healthy donors and patients with cystic fibrosis (CF) (7–14), suggesting that patients with CF are not defective in NET formation. Even though NET formation is beneficial for the host by controlling bacterial spreading, excessive NET formation is toxic to lung epithelium and vascular endothelium (15, 16); NETs have detrimental effects on vascular and endothelial permeability, thereby causing lung tissue damage and ARDS (17, 18). Furthermore, during inflammatory responses, activated human neutrophils and epithelial cells (EPCs) secrete the antimicrobial peptide LL-37, a member of the cathelicidin family that facilitates NET formation (19). Similarly, mouse neutrophils secrete cathelicidin-related antimicrobial peptide (CRAMP), the ortholog of human LL-37, during inflammatory reactions (20). Impaired bacterial clearance and delayed neutrophil influx were observed in the lungs of CRAMP-deficient mice infected with *Klebsiella pneumoniae* (21), and exogenous LL-37 augments clearance of pulmonary *P*. *aeruginosa* in a mouse model by promoting an early neutrophil response (22). Thus, we wanted to better understand how LL-37/CRAMP contributes to host defense against *P*. *aeruginosa* invasion.

NET formation can be induced by signals that activate NADPH oxidase–dependent and –independent pathways (23). It has been reported that phorbol 12-myristate 13-acetate (PMA) activates NADPH oxidase, thus triggering elastase release, cytoskeleton degradation, and gasdermin D (GSDMD) cleavage (24). Furthermore, cytosolic LPS and cytosolic Gram-negative bacteria (*Salmonella* deficient of SifA protein [*Salmonella* ΔsifA] and *Citrobacter rodentium*) activate noncanonical (caspase-4/11) inflammasome signaling and trigger GSDMD-dependent neutrophil death (25). In addition to neutrophils, LPS can activate caspase-1 and caspase-4/5/11 via canonical and noncanonical pathways, respectively, thereby cleaving GSDMD to induce pyroptotic cell death in macrophages (26, 27). All of these observations suggest that GSDMD plays a critical role in inflammasome-mediated cell death and IL-1β release.

CLEC5A is a pivotal pattern recognition receptor (PRR) in host defense against viral (28–30) and Gram-positive bacterial infections (31). CLEC5A is expressed by monocytes, neutrophils, and macrophages but not by lymphocytes or nonimmune cells. Activation of CLEC5A triggers spleen tyrosine kinase–dependent (Syk-dependent) NALP3 inflammasome activation, caspase-1–dependent IL-1β production (29), ROS production, NET formation, and proinflammatory cytokine secretion (31). Moreover, *Clec5a^–/–^* neutrophils are impaired in producing NET and proinflammatory cytokine production upon engagement with *Listeria monocytogenes*, and *Clec5a^–/–^* mice are highly susceptible to *L*. *monocytogenes* infection (31). These observations suggest that CLEC5A is involved in host defense against microbial infections. To further understand this, we previously used a CLEC5A.Fc fusion protein to probe a bacterial glycan array in order to identify potential pathogen-associated ligands and found that CLEC5A.Fc binds glycans derived from *P*. *aeruginosa* (31).

Here, we report that *P*. *aeruginosa* (PAO1 strain) engages CLEC5A to induce NET formation and proinflammatory cytokine release. While CLEC5A is responsible for *P*. *aeruginosa*–induced caspase-1–dependent NET formation, it does not contribute to GSDMD cleavage and pyroptosis in neutrophils. In contrast, CLEC5A is critical for *P*. *aeruginosa*–induced proinflammatory cytokine release and GSDMD cleavage in macrophages. Blockade of CLEC5A not only attenuates lung inflammation and epithelial damage, but also it increases the efficacy of ciprofloxacin and prevents lung fibrosis caused by *P*. *aeruginosa*. Thus, blockade of CLEC5A is a promising strategy to reduce the risk of ARDS in patients who have suffered from *P*. *aeruginosa* infection.

## Results

### CLEC5A mediates P. aeruginosa–induced NET formation via caspase-1.

**Figure 1:**
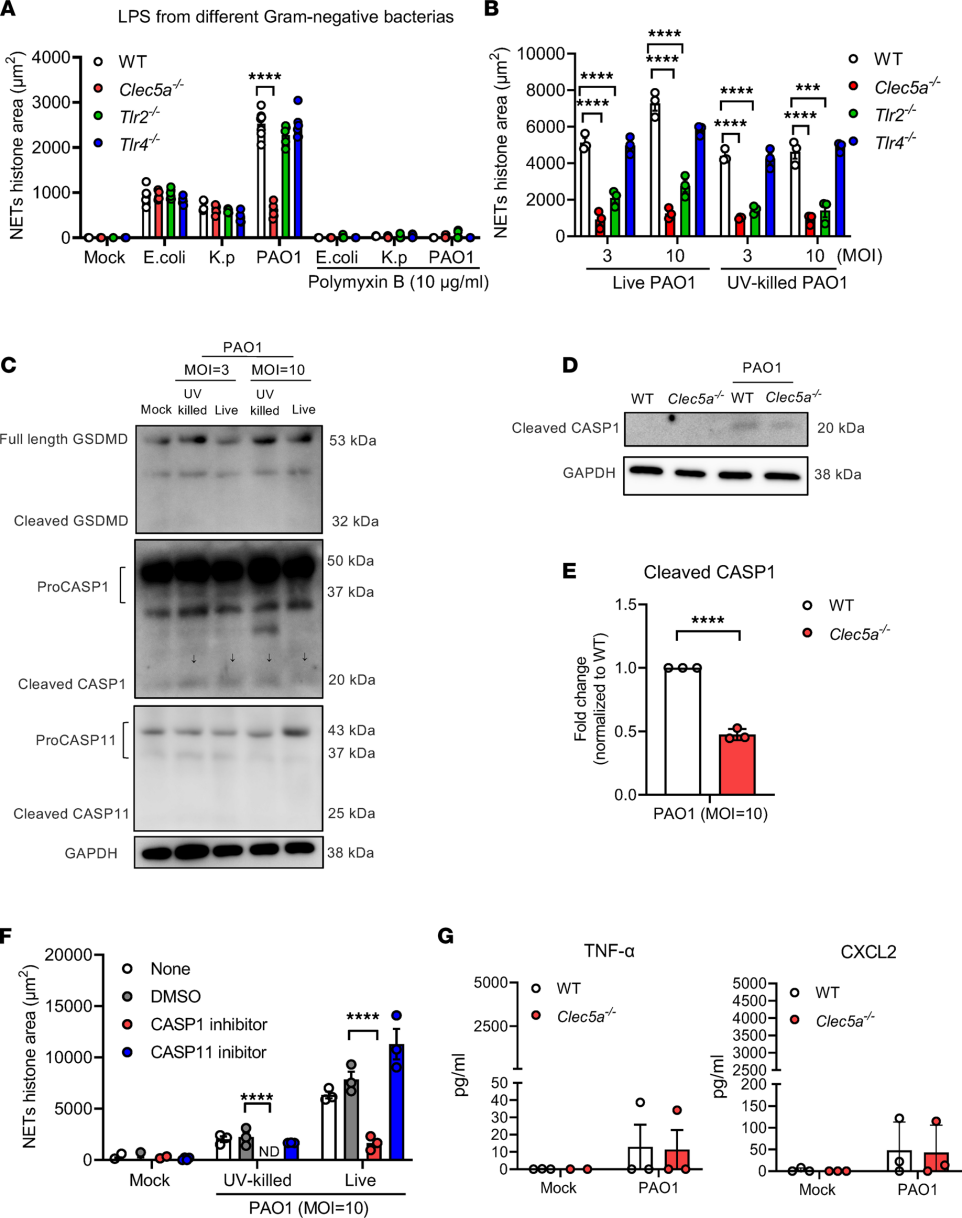
*P*. *aeruginosa* induces caspase-1–dependent NET formation via CLEC5A. (**A** and **B**) Neutrophils from WT, *Clec5a^–/–^*, *Tlr2^–/–^*, and *Tlr4^–/–^* mice were stimulated with LPS from *E*. *coli, Klebsiella pneumoniae* (*K.p*), and *P*. *aeruginosa* PAO1 (10 μg/mL) in the presence or absence of the antibiotic polymyxin B (10 μg/mL) (*n* = 5 independent experiments) (**A**), or live and UV-killed *P*. *aeruginosa* PAO1 (MOI = 3 or 10) at 37°C for 1 hour (*n*= 3 independent experiments) (**B**). (**C**) Mouse BM-derived neutrophils (5 × 10^5^/mL) were incubated with live PAO1 or UV-killed PAO1 (MOI = 3 or 10) for 1 hour at 37°C. For inflammasome activation, neutrophils were primed with LPS from *E*. *coli* (0.5 μg/mL) for 3 hours and then stimulated with ATP (3 mM) or nigericin (10 μM) for 45 minutes at 37°C. Western blots of cell lysates (20 μg) were probed with anti-GSDMD Ab, anti–caspase-1(CASP1) p20 Ab, anti–caspase-11 (CASP11) Ab, or anti-GAPDH Ab. Arrows indicate the location of caspase-1 p20. (**D** and **E**) Neutrophils from WT and *Clec5a^–/–^* mice were incubated with PAO1 (MOI = 10) for 1 hour at 37°C; caspase-1 (CASP1) cleavage was determined by western blotting (**D**), and images (*n* = 3) were quantified using ImageJ (NIH) software. Western blot analyses were repeated for 3 times (**E**). (**F**) WT neutrophils were preincubated with DMSO, caspase-1 inhibitor Z-WEHD-FMK (20 μM), or caspase-11 inhibitor wedelolactone (100 μM) for 1 hour at room temperature, and they were further incubated with UV-killed or live PAO1 (MPO = 10) for 1 hour at 37°C (*n* = 3 independent experiments). The level of NETs was calculated using the area (μm^2^) of histone overlapping with MPO by MetaMorph software. (**G**) Cytokine/chemokine in culture media was determined by ELISA. Data are presented as mean ± SEM. Statistical test for **A** and **B** were measured with 2-way ANOVA, and **E**–**G** were calculated with an unpaired and nonparametric Student’s *t* test with Mann-Whitney *U* test. ****P < 0.0001.

LPS from various bacterial strains have been shown to induce NET formation (32). To understand the roles of CLEC5A in *P*. *aeruginosa* infection, we isolated neutrophils from WT, *Clec5a^–/–^*, *Tlr2^–/–^*, and *Tlr4^–/–^* mice and compared their responses with various LPS. NET formation was determined by the colocalization of histone, myeloperoxidase (MPO), and DNA under a confocal microscope (Supplemental Figure 1, A and B; supplemental material available online with this article; https://doi.org/10.1172/jci.insight.156613DS1). Surprisingly, the levels of *E*. *coli* LPS-induced NET formation were similar in WT, *Clec5a^–/–^*, *Tlr2^–/–^*, and *Tlr4^–/–^* neutrophils (Figure 1A). A similar observation was found in neutrophils incubated with LPS from *K. pneumoniae* (Figure 1A). However, NET formation induced by LPS from the *P*. *aeruginosa* PAO1 strain was attenuated in *Clec5a^–/–^* neutrophils (Figure 1A). These observations suggest that LPS from PAO1 induces NET formation via CLEC5A. In contrast, phagocytosis and killing of *P*. *aeruginosa* in neutrophils were unaffected by *Clec5a* deletion (Supplemental Figure 1, C and D). We further compared NET formation in neutrophils incubated with live and UV-inactivated *P*. *aeruginosa*; while both live and UV-inactivated *P*. *aeruginosa* induced similar levels of NET formation in WT and *Tlr4^–/–^* mice, NET formation was reduced dramatically in *Clec5a^–/–^* and *Tlr2^–/–^* neutrophils (Figure 1B). These observations suggest that *P*. *aeruginosa*–induced NET formation occurs via CLEC5A and TLR2 and is not dependent on toxic substances secreted from live bacteria. TLR2 does not bind *P*. *aeruginosa* LPS; therefore, the less potent effect of TLR2 deletion compared with *Clec5a* deletion may be due to the coactivation of the CLEC5A/TLR2 complex by *P*. *aeruginosa* as we observed in *L. monocytogenes*-induced NET formation (31).

Even though both live and UV-killed *P*. *aeruginosa* activated caspase-1, we observed neither caspase-11 activation nor GSDMD cleavage in response to *P*. *aeruginosa* PAO1 (Figure 1C). Furthermore, *P*. *aeruginosa–*induced caspase-1 cleavage was attenuated in *Clec5a^–/–^* neutrophils (Figure 1, D and E). We further found that NET formation was inhibited by caspase-1 inhibitor but not caspase-11 inhibitor (Figure 1F). Low levels of TNF-α and CXCL2 were detected in both WT and *Clec5a^–/–^* neutrophils in response to *P*. *aeruginosa* PAO1 (Figure 1G), while the other cytokines (IL-6, IL-1β, and CXCL1) were not detectable (data not shown). These observations suggest that CLEC5A is critical in *P*. *aeruginosa*–induced canonical inflammasome activation and caspase-1–dependent NET formation but that it does not contribute significantly to proinflammatory cytokine release from neutrophils (29, 31).

### CLEC5A is critical for P. aeruginosa–induced inflammatory cytokine release by macrophages.

**Figure 2:**
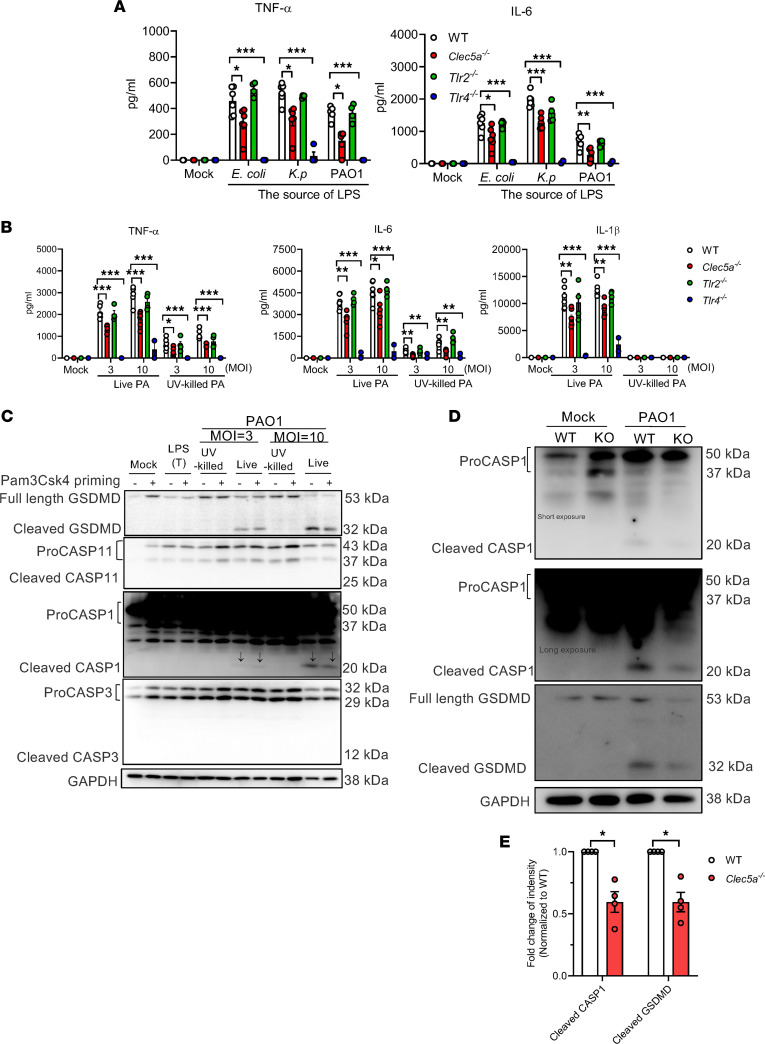
*P*. *aeruginosa* induces cytokine production and caspase-1–dependent GSDMD cleavage in BM-derived macrophages. (**A** and **B**) BMDMs from WT (*n* = 6), *Clec5a^–/–^* (*n* = 6), *Tlr2^–/–^* (*n* = 4), and *Tlr4^–/–^* (*n* = 4) mice were stimulated with LPS from *E.coli, Klebsiella pneumoniae* (*K.p*), and PAO1 (100 ng/mL) (**A**), or they were incubated with live or UV-killed *P*. *aeruginosa* PAO1 strain (MOI = 3 or 10) (**B**) at 37°C for 1 hour. After washing, with serum-free RPMI containing 10 μg/mL gentamicin, cells were cultured in fresh RPMI containing 10% (v/v) FBS for 24 hours at 37°C. The levels of cytokine were measured by ELISA. Data were collected from 6 independent experiments. (**C**) BMDMs from WT mice were primed with Pam3CsK4 (1 μg/mL) for 4 hours at 37°C, followed by incubation with UV-killed or live *P*. *aeruginosa* PAO1 strain (MOI = 3 or 10) or transfected (T) with LPS from *E*. *coli* (2.5 μg/mL) for 15 hours at 37°C. Western blots were incubated with anti-GSDMD Ab, anti–caspase-11 (CASP11) Ab, anti–caspase-1 (CASP1) Ab, anti–caspase-3 (CASP3) Ab, or anti-GAPDH Ab. (**D** and **E**) BMDMs from WT and *Clec5a^–/–^* mice were incubated with live *P*. *aeruginosa* PAO1 strain (MOI = 10) for 15 hours at 37°C. Western blots of cell lysates were probed with anti-GSDMD Ab, anti–caspase-1 Ab, or anti-GAPDH Ab. Cleaved caspase-1 (cleaved CASP1) and cleaved GSDMD are shown as fold change compared with WT. LPS (T), transfection of *E*. *coli* LPS. Representative immunoblot of 4 independent experiments. Data are mean ± SEM, and statistical analysis for **A** and **B** were performed with 2-way ANOVA and by unpaired and nonparametric Student’s *t* test with Mann-Whitney *U* test for **E**. *P < 0.05, **P < 0.01, ***P < 0.001.

We further asked whether CLEC5A has any effect on LPS-induced proinflammatory cytokine release by BM-derived macrophages (BMDM). While proinflammatory cytokine release was almost abolished in *Tlr4^–/–^* BMDMs after stimulation with LPS from various Gram-negative bacteria, the levels of TNF-α and IL-6 were attenuated mildly in *Clec5a^–/–^* BMDMs (Figure 2A). It is interesting to note that live *P*. *aeruginosa* had more potent effects than LPS and UV-inactivated *P*. *aeruginosa* as an inducer of proinflammatory cytokine release (Figure 2B). This observation is in accordance with the previous report that *P*. *aeruginosa* exoenzyme S is responsible for the induction of proinflammatory cytokines and chemokines (33), while the type III secretion system is responsible for activation of caspase-1 and IL-1β production (34). We found that LPS (from *E*. *coli*) induced mild GSDMD cleavage and caspases-11 activation, but not caspase-1 or caspase-3 in transfected BMDMs (Figure 2C, lines 3–4 from left). However, *P*. *aeruginosa* induced both GSDMD cleavage and caspase-1 activation in WT BMDMs, while neither caspase-3 nor caspase-11 was activated under the same conditions (Figure 2C and Supplemental Figure 2). In contrast to WT neutrophils, caspase-1 activation and GSDMD cleavage were attenuated significantly in *Clec5a^–/–^* BMDMs (Figure 2, D and E). Thus, CLEC5A is critical in *P*. *aeruginosa*–induced proinflammatory cytokine release and caspase-1–dependent GSDMD cleavage in BMDMs.

### P. aeruginosa induces neutrophil infiltration and CRAMP release via CLEC5A.

**Figure 3:**
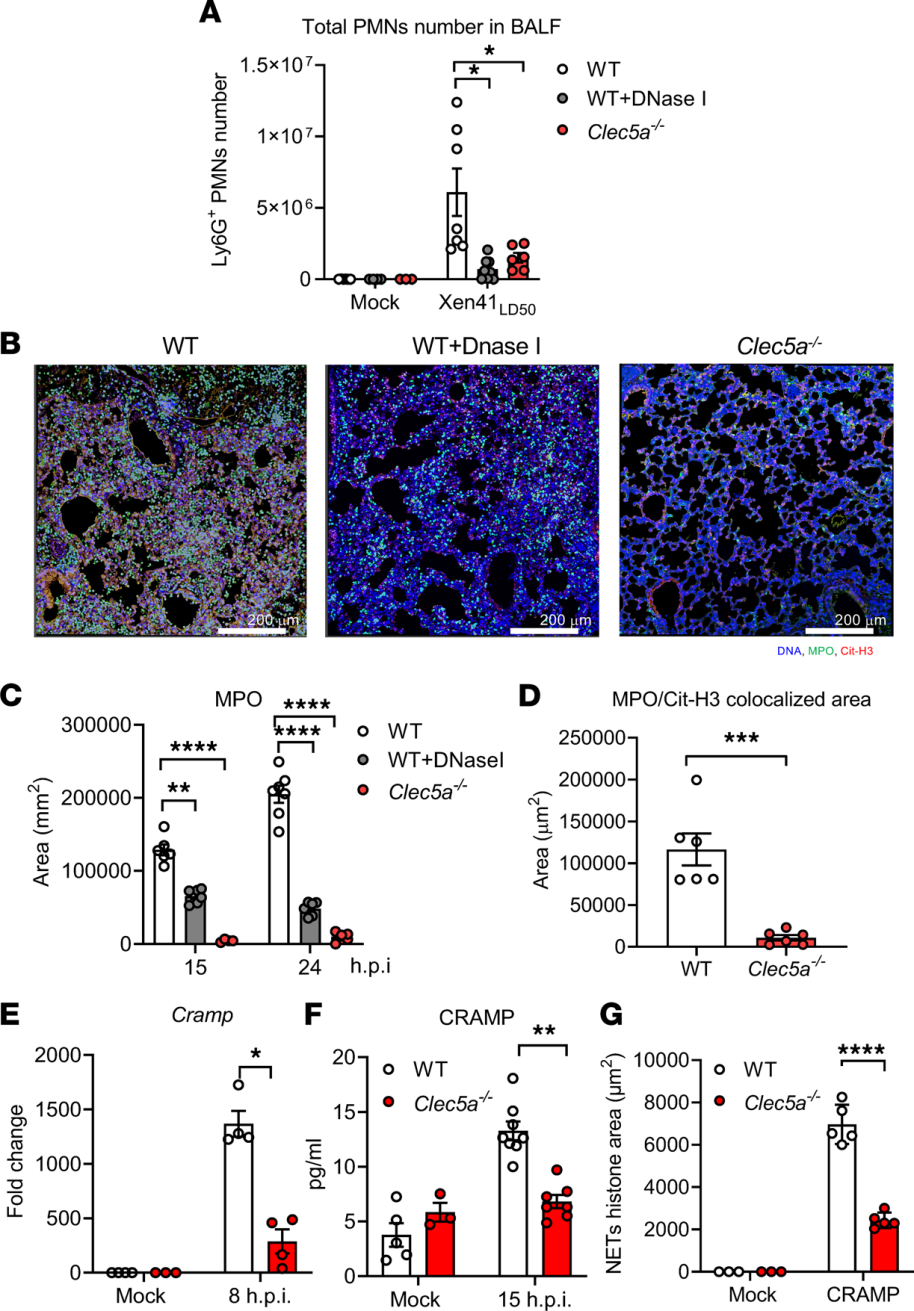
*P*. *aeruginosa* upregulates CRAMP to promote NET formation via CLEC5A in vivo. (**A**) WT and *Clec5a^–/–^* mice were intratracheally inoculated with median lethal dose (LD_50_) of *P*. *aeruginosa* (Xen41, PAO1 strain), and BALF was harvested at 15 hours after infection to detect Ly6G^+^ cells (mock: WT, *n* = 6; *Clec5a^–/–^, n* = 3; WT + DNase 1, *n* = 4; Xen41_LD50_: WT, *n* = 7; *Clec5a^–/–^, n* = 6; WT + DNase 1, *n* = 8). (**B**) To detect NET formation, lung tissue was collected at 24 hours after infection and fixed in 10% formamide and embedded in paraffin. Tissue sections were stained with antibodies to myeloperoxidase (MPO) (green), citrullinated histone H3 (Cit-H3) (red), and Hoechst 33342 (blue) for NET structure visualization. Scale bar: 200 μm. (**C** and **D**) The areas of MPO (**C**) and MPO/Cit-H3 colocalization (**D**) were analyzed using MetaMorph software (*n* = 6 for each group). (**E**) Expression levels of *Cramp* mRNA in tissues were determined by qPCR (mock: WT, *n* = 4; *Clec5a^–/–^,* *n* = 3; Xen41_LD50_: WT, *n* = 4; *Clec5a^–/–^*, *n* = 4). (**F**) CRAMP protein in BALF was measured by ELISA (mock: WT, *n* = 5; *Clec5a^–/–^, n* = 3; Xen41_LD50_: WT, *n* = 8; *Clec5a^–/–^, n* = 7) (**F**). *P < 0.05, **P < 0.01, ***P < 0.001, ****P < 0.0001 (2-way ANOVA). (**G**) Neutrophils from WT and *Clec5a^–/–^* mice were stimulated with CRAMP (50 μg/mL) at 37°C for 3 hours (mock: WT, *n* = 3; *Clec5a^–/–^*, *n* = 3; Xen41_LD50_: WT, *n* = 5; *Clec5a^–/–^, n* = 5). The level of NET formation was calculated from the area (μm^2^) of histone overlapping with MPO using MetaMorph software. Data are mean ± SEM from at least 3 independent experiments. The statistical significance was calculated with 2-way ANOVA for **A** and **C** and by unpaired and nonparametric Student’s *t* test with Mann-Whitney *U* test for **D**–**G**. *P < 0.05, **P < 0.01, ***P < 0.001, ****P < 0.0001.

We further examined the role of CLEC5A in *P*. *aeruginosa*–induced NET formation in vivo. We used a luciferase-expressing PAO1 strain (Xen41) in order to track the migration of *P*. *aeruginosa* after inoculation of WT and *Clec5a^–/–^* mice. Mice were inoculated with a median lethal dose (LD_50_) of *P*. *aeruginosa* Xen41, followed by determination of PMN infiltration into the lungs (quantified in BALF) and detection of NET formation (staining for DNA [blue], MPO [green], citrullinated histone H3 [Cit-H3, red]) after 24 hours. Compared with WT mice, *P*. *aeruginosa*–induced Ly6G^+^ cell infiltration (Figure 3A) and NET formation (Figure 3B and Supplemental Figure 3) were attenuated in both DNase I–treated and *Clec5a^–/–^* mice. Since DNase I efficiently destroys NET structure, this observation suggests that NET formation was a feature of a significant proportion of the cells for which infiltration was suppressed in the lungs of *Clec5a^–/–^* mice. Moreover, MPO (Figure 3C) and colocalized MPO/Cit-H3 (Figure 3D) were also significantly reduced in *Clec5a^–/–^* mice. We tracked the distribution of *P*. *aeruginosa* using the IVIS spectrum in vivo imaging system (Supplemental Figure 4A). In WT mice, luminescence signal was observed in the lung at 24 hours after inoculation and reached a peak at 48 hours after inoculation (Supplemental Figure 4B). In contrast, luminescence signal barely rose above baseline in *Clec5a^–/–^* mice (Supplemental Figure 4B). We further investigated bacterial loads in the visceral organs after inoculation with *P*. *aeruginosa*. In WT mice, higher bacterial loads were found in lung and liver (Supplemental Figure 4C), peaking at 24 hours after infection. In contrast, *Clec5a^–/–^* mice had higher bacterial loads in blood, kidney, and spleen (Supplemental Figure 4D), peaking at 15 hours. These observations suggest that reduced NET formation is associated with reduced neutrophil infiltration to the lungs in *Clec5a^–/–^* mice and that CLEC5A has a major role in controlling *P*. *aeruginosa* distribution after intratracheal inoculation.

Since upregulation of the human cathelicidin antimicrobial peptide LL-37 has been observed during lung inflammation (35), and LL-37 has been reported to facilitate NET formation (19), we asked whether CLEC5A contributed to upregulation of CRAMP in mice. While we observed that *P*. *aeruginosa* induced high levels of *Cramp* mRNA in the lung (Figure 3E) and CRAMP protein in BALF (Figure 3F) in WT mice, CRAMP expression was attenuated dramatically in *Clec5a^–/–^* mice (Figure 3, E and F), suggesting that CLEC5A was involved in *P*. *aeruginosa*–induced CRAMP release. Moreover, CRAMP-induced NET formation was attenuated dramatically in *Clec5a^–/–^* neutrophils (Figure 3G), suggesting that CLEC5A has a significant role in this process. Thus, CLEC5A deficiency not only impairs CRAMP release, but also attenuates *P*. *aeruginosa–* and CRAMP-induced NET formation.

### CLEC5A is critical in P. aeruginosa–induced lung inflammation.

**Figure 4:**
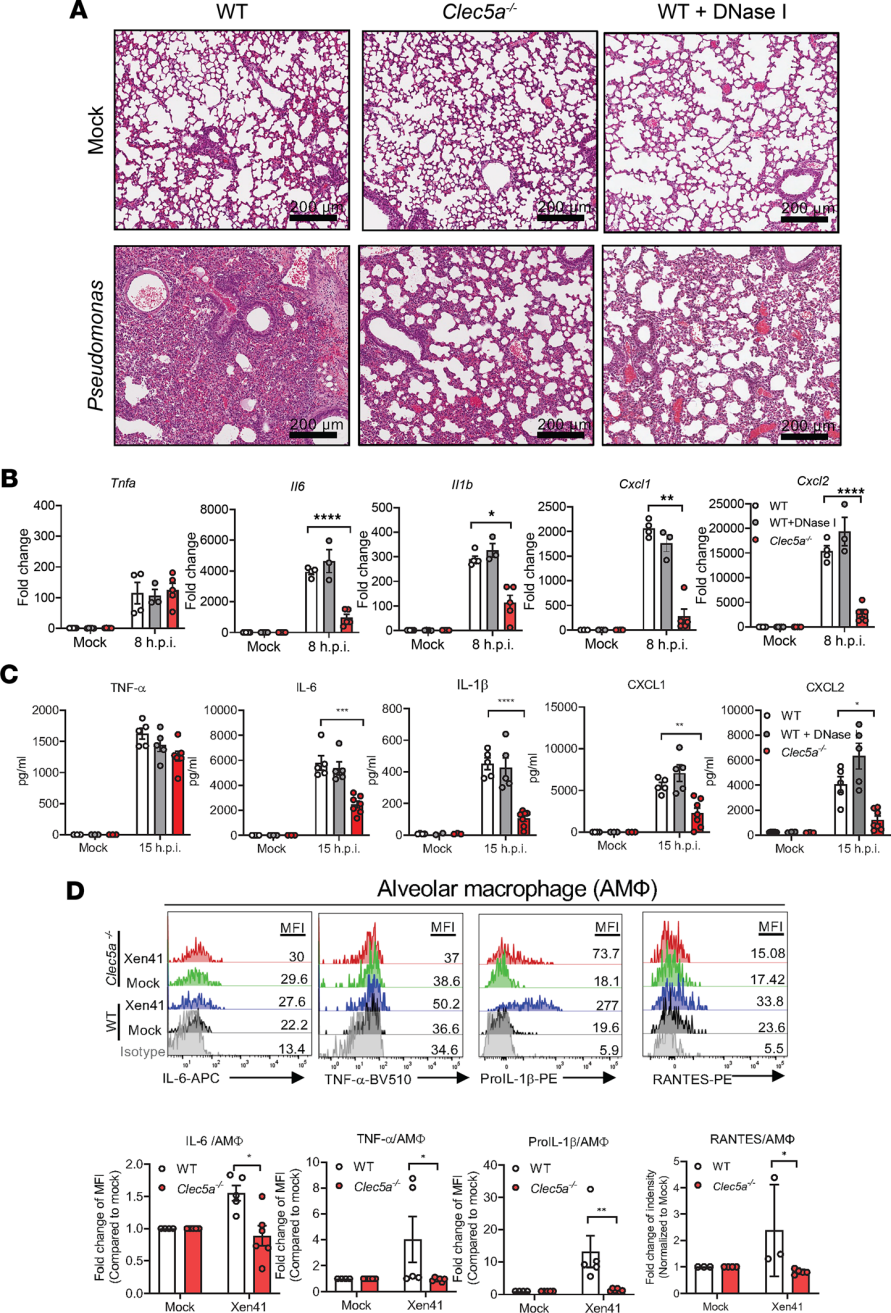
CLEC5A is critical for *P*. *aeruginosa*–induced cell infiltration and inflammation in the lung. WT and *Clec5a^–/–^* mice were intratracheally inoculated with median lethal dose (LD_50_) *of P*. *aeruginosa* (Xen41, PAO1 strain); WT mice were injected with DNase I (4 U/mouse) via i.p. route for comparison. (**A**) Lung tissue was collected at 24 hours after infection for H&E staining. Scale bar: 200 μm. (**B**) Lung tissue was harvested at 8 hours after infection for measurement of cytokine and chemokine mRNA levels. Data are mean ± SEM (mock: WT, *n* = 6; WT + DNase 1, *n* = 5; *Clec5a^–/–^, n* = 5; Xen41_LD50_: WT, *n* = 4; WT + DNase 1, *n* = 3; *Clec5a^–/–^, n* = 5). (**C**) BALF was collected at 15 hours after infection, and cytokines and chemokines were quantified by ELISA. Data are mean ± SEM (mock: WT, *n* = 3; WT + DNase 1, *n* = 3; *Clec5a^–/–^*, *n* = 3; Xen41_LD50_: WT, *n* = 5; WT + DNase 1, *n* = 5; *Clec5a^–/–^*, *n* = 7). (**D**) Lung cell suspensions from WT and *Clec5a^–/–^* mice were stained with PE/Cy7-conjugated anti-CD45 Ab, PerCP/Cy5.5-conjugated anti-CD64 Ab, APC/Cy7-conjugated anti-IA/IE Ab, BV421-conjugated anti–Siglec-F Ab, BV510-conjugated anti–TNF-α Ab, PE-conjugated anti–pro–IL1β Ab, APC-conjugated anti–IL-6 Ab, PE-conjugated anti-RANTES Ab to measure intracellular TNF-α, IL-6, pro–IL-1β, and RANTES in alveolar macrophage (AMФ: CD45^+^CD64^+^IA/IE^+^Siglec-F^+^). Cytokine/chemokine MFIs were normalized to mock for WT and *Clec5a^–/–^* mice, and the data (fold change of MFI) are presented as mean ± SEM (mock: WT, *n* = 3; *Clec5a^–/–^, n* = 3; Xen41_LD50_: WT, *n* = 4; *Clec5a^–/–^, n* = 4). Statistical calculations were performed with 2-way ANOVA. *P < 0.05, **P < 0.01, ***P < 0.001, ****P < 0.0001.

Since NETs stimulate lung EPCs to induce proinflammatory responses (36), we asked whether CLEC5A contributes to cell infiltration and lung inflammation after *P*. *aeruginosa* infection. While extensive neutrophil infiltration was observed in WT mice (Figure 4A, left lower panel), this was attenuated in *Clec5a^–/–^* mice (Figure 4A, middle lower panel) and DNase I–treated WT mice (Figure 4A, right lower panel). This observation further suggests that NET formation contributes to *P*. *aeruginosa*–induced neutrophil infiltration into the lung. We examined the expression of proinflammatory cytokine and chemokine mRNAs in lung tissue by real-time PCR at 8 hours after infection (Figure 4B) and harvested BALF to determine cytokine/chemokine levels by ELISA at 15 hours after infection (Figure 4C). Compared with WT mice, the expression of *Il6, Il1b, Cxcl1,* and *Cxcl2* mRNAs was downregulated in *Clec5a^–/–^* mice (Figure 4B). A similar phenomenon was observed with regard to protein levels in BALF (Figure 4C). We further examined the expression of CLEC5A and cytokines in alveolar macrophages (AMФ; Supplemental Figure 5) from *P*. *aeruginosa*–inoculated mice by intracellular staining. We found that inoculation of *P*. *aeruginosa* upregulated the expression of CLEC5A (Supplemental Figure 5) and proinflammatory cytokines and chemokines (IL-6, TNF-α, pro–IL-1β, and RANTES) in WT AMФ; we also found that the expression of all 4 proteins was downregulated in *Clec5a^–/–^*AMФ (Figure 4D). We tested whether *P*. *aeruginosa* induced cytokine and chemokine production in neutrophils (Supplemental Figure 6) and alveolar EPCs (Supplemental Figure 7) and found that WT neutrophils expressed low levels of cytokines and chemokines, and CLEC5A deficiency further reduced pro–IL-1β expression in vivo (Supplemental Figure 6). Furthermore, the production of TNF-α and pro–IL-1β were attenuated in *Clec5a^–/–^* EPCs, even though EPCs lack detectable expression of CLEC5A (Supplemental Figure 7). Thus, we conclude that CLEC5A contributes to cell infiltration, proinflammatory cytokine/chemokine release, and lung inflammation after *P*. *aeruginosa* infection in vivo.

### CLEC5A mediates P. aeruginosa–induced pulmonary vascular permeability change and lethality.

**Figure 5:**
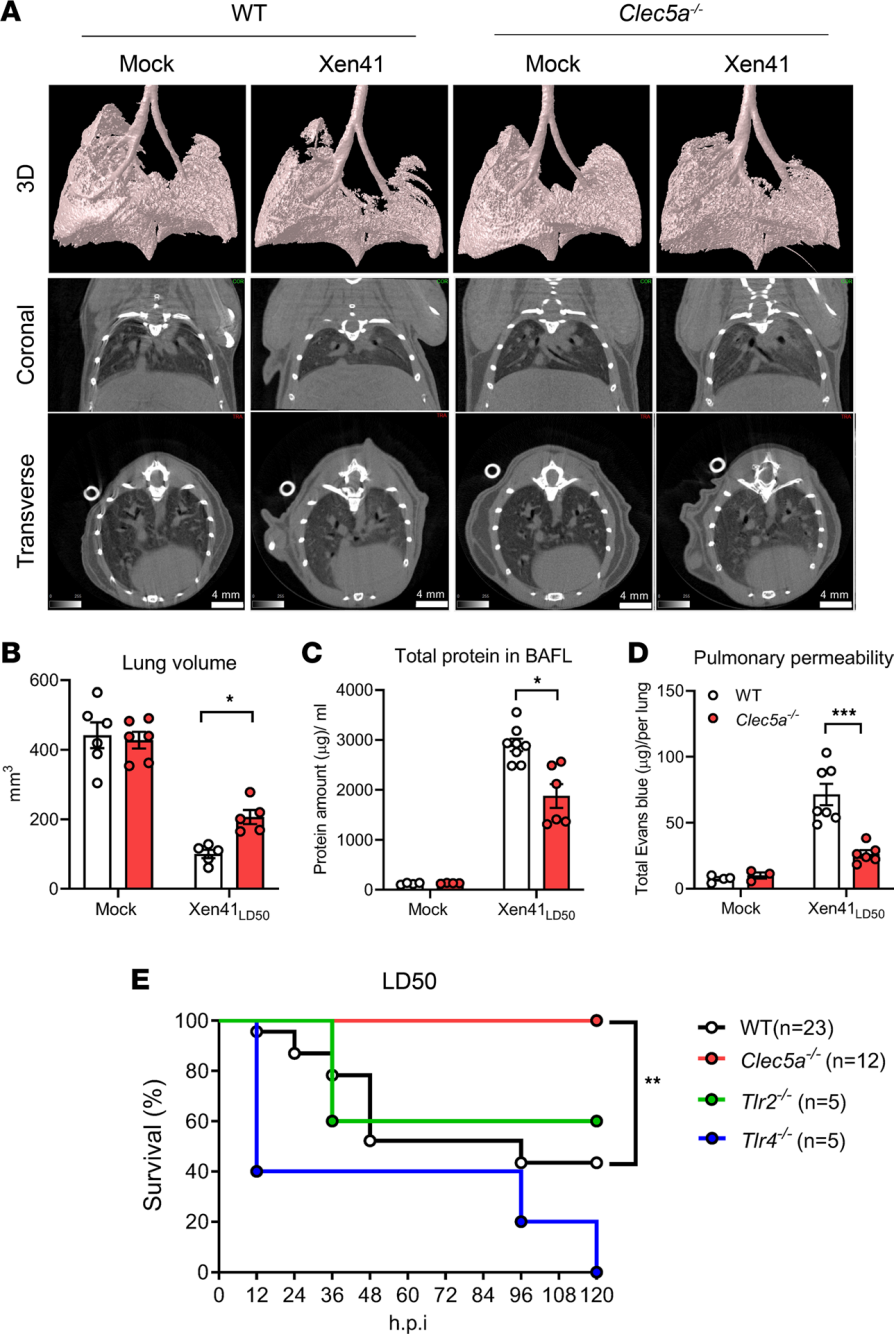
CLEC5A mediates *P*. *aeruginosa*–induced lung injury and inflammation. WT and *Clec5a^–/–^* mice were intratracheally injected with *P*. *aeruginosa* Xen41 at median lethal dose (LD_50_). (**A**) Three-dimensional images of lungs were reconstructed, and coronal and transverse images were captured using a μCT scanner (SkyScan1276, with CTAn software) at 15 hours after infection. Scale bar: 4 mm. (**B**) Lung volume was calculated using the CTAn software. Data are mean ± SEM (mock: WT, *n* = 6; *Clec5a^–/–^*, *n* = 6; Xen41_LD50_: WT, *n* = 5; *Clec5a^–/–^, n* = 5). (**C**) BALF was collected at 15 hours after infection, and the protein content was determined using the DC protein assay kit. Data are mean ± SEM (mock: WT, *n* = 4; *Clec5a^–/–^, n* = 4; Xen41_LD50_: WT, *n* = 8; *Clec5a^–/–^, n* = 7). (**D**) Evans Blue (0.1%, 150 μL) was i.v. injected into mice at 14 hours after infection. Lung tissue was harvested after perfusion with 20 mL of PBS, and samples were heated in 2 mL of formamide to extract the accumulated Evans Blue after 1 hour of circulation. Data are mean ± SEM (mock: WT, *n* = 4; *Clec5a^–/–^*, *n* = 4; Xen41_LD50_: WT, *n* = 7; *Clec5a^–/–^*, *n* = 10). (**E**) WT (*n* = 23), *Clec5a^–/–^* (*n* = 12), *Tlr2^–/–^*(*n* = 5), and *Tlr4^–/–^* (*n* = 5) mice were intratracheally challenged with LD_50_ of *P*. *aeruginosa* Xen41. Survival rate was monitored until 120 hours after infection. Survival rate was assessed using the log-rank (Mantel-Cox) test with GraphPad Prism software. Data are mean ± SEM and calculated the statistical significance with 2-way ANOVA. *P < 0.05, **P < 0.01, ***P < 0.001.

We went on to explore whether CLEC5A contributes to increased vascular permeability and lethality after *P*. *aeruginosa* infection. To address this question, mice were challenged with LD_50_ of *P*. *aeruginosa* (Xen41 strain) by an intratracheal route to evaluate lung inflammation and measure lung volume at 15 hours after infection by μCT (Figure 5, A and B), and pulmonary vascular permeability was assessed by measuring total protein concentration in BALF (Figure 5C) and measuring accumulation of Evans Blue in the lungs following i.v. administration (Figure 5D). In the 3-dimensional reconstruction images (Figure 5A), lung volume was seen to be dramatically reduced in WT mice (23% of mock-infected mice) after infection, indicating that alveolar space becomes constricted after infection. While lung volume was reduced in *Clec5a^–/–^* mice after infection, it was much higher than in WT mice (Figure 5, A and B). Compared with WT mice, we detected significantly reduced protein concentration in BALF (Figure 5C) and reduced Evans Blue accumulation in lung extracts (Figure 5D) from *Clec5a^–/–^* mice following *P*. *aeruginosa* infection. These observations are in accord with attenuated lung inflammation in CLEC5A-deficient mice (Figure 4A). We further compared the survival rates of *Clec5a^–/–^*, WT, and TLR-deficient mice after challenge with LD_50_ of *P*. *aeruginosa*. While the survival rates of WT and *Tlr2^–/–^* mice were similar, all the *Tlr4^–/–^* mice died by 120 hours after infection. In contrast, all the *Clec5a^–/–^* mice survived for 120 hours following *P*. *aeruginosa* infection (Figure 5E). These observations further demonstrate the critical role of CLEC5A in *P*. *aeruginosa*–induced changes in lung vascular permeability and lethality.

### Anti-CLEC5A mAb protects mice from P. aeruginosa–induced lung damage.

**Figure 6:**
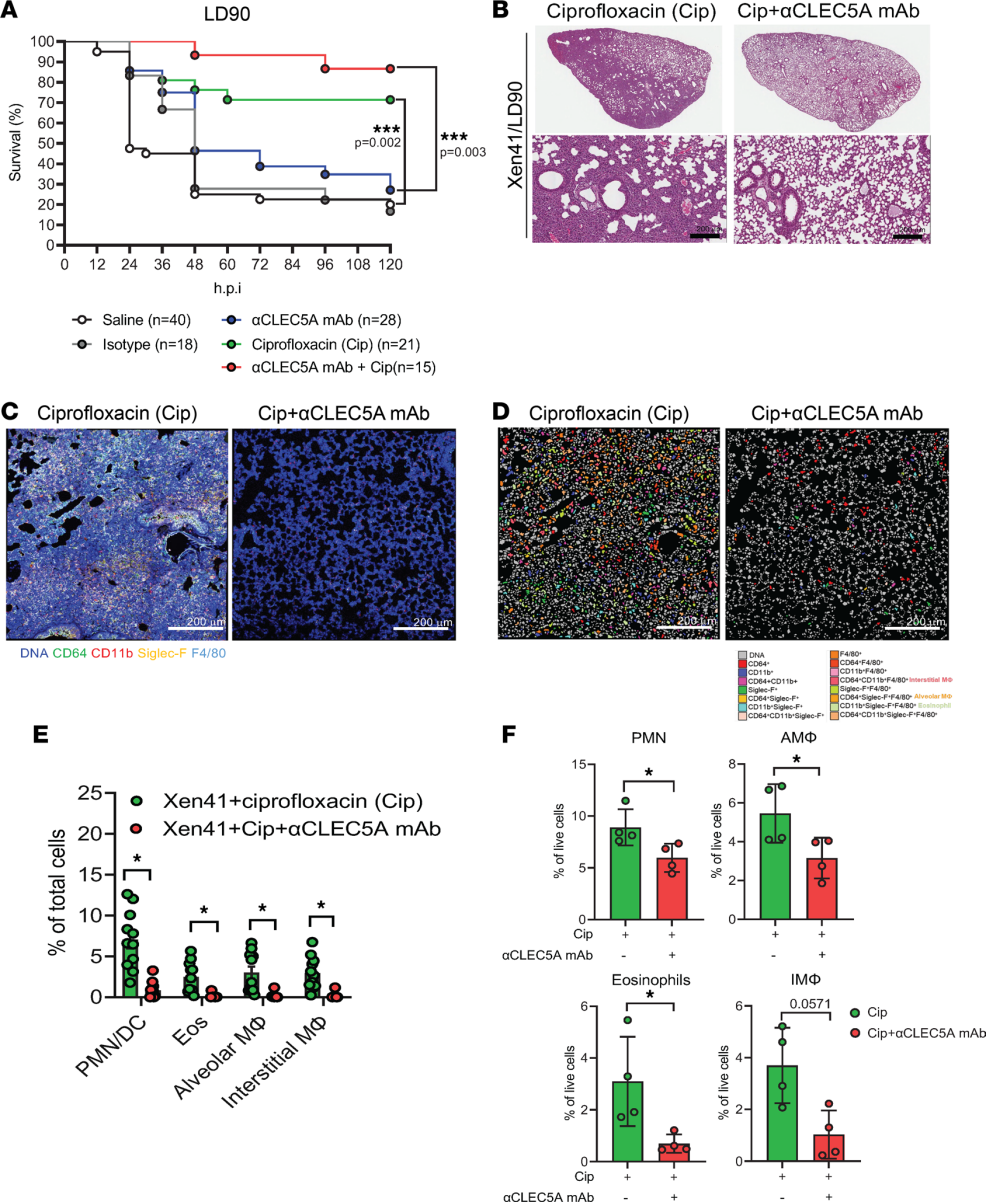
CLEC5A is a potential therapeutic target in *P*. *aeruginosa*–induced cell infiltration and lethality. WT mice were intratracheally injected with a sublethal dose (LD_90_) of *P*. *aeruginosa* Xen41 (1 × 10^6^ PFU) and injected with isotype control (mIgG1, 100 μg per mouse, i.v.), anti-mCLEC5A mAb (clone 4A12D5, 100 μg per mouse, i.v.), ciprofloxacin (Cip, 4 mg/kg, i.p.), or anti-mCLEC5A mAb (i.v.) combined with ciprofloxacin (i.p.) at 15 hours after infection. Ciprofloxacin was further administered every 12 hours. (**A**) Survival rate was monitored up to 120 hours after infection. (**B**) Lung tissues were collected, and formalin-fixed paraffin-embedded sections were prepared. Cellular infiltration was visualized by H&E staining. Scale bar: 200 m. (**C**) Tissue sections were stained with anti-CD64 Ab (green), anti-CD11b Ab (red), anti–Siglec-F Ab (yellow), and anti-F4/80 Ab (light blue); fluorescence images were captured using a Leica confocal microscope with white light laser system (TCS-SP8-MP-SMD) and analyzed using Leica Application Suite X software. Scale bar: 200 μm. (**D** and **E**) The cell population was analyzed with MetaMorph software to determine the percentages of interstitial macrophages (interstitial MФ, CD64^+^CD11b^+^Siglec-F^–^F4/80^+^), alveolar macrophages (AMФ, CD64^+^CD11b^–^Siglec-F^+^F4/80^+^), eosinophils (Eos, CD64^–^CD11b^+^Siglec-F^+^F4/80^+^), and neutrophils/DCs (PMN/DC, CD11b^+^Siglec-F^–^F4/80^–^CD64^–^) (ciprofloxacin, *n* = 11; ciprofloxacin + anti-mCLEC5A mAb, *n* = 10, from 4 independent experiments) (**F**) Lung cell suspensions were incubated with BV650-conjugated anti-Ly6G Ab, BUV805-conjugated anti-CD45 Ab, BUV395-conjugated anti-CD11b Ab, BV480-conjugated anti-CD11c Ab, BV605-conjugated anti-CD64 Ab, PE-Cy5-conjugated anti-CD25 Ab, and BUV496-conjugated anti-IA/IE Ab to analyze the percentage of neutrophil (PMN), alveolar macrophage (AMФ), interstitial macrophage (IMФ), and eosinophils after *P*. *aeruginosa* inoculation (ciprofloxacin, *n* = 4; ciprofloxacin + anti-mCLEC5A mAb, *n* = 4, from 3 independent experiments). Survival rate was assessed using the log-rank (Mantel-Cox) test with GraphPad Prism software. ***P < 0.005. Statistical analysis for **D** and **E** was calculated with unpaired and nonparametric Student’s *t* test with Mann-Whitney *U* test. *P < 0.05.

Anti-CLEC5A mAb has been shown to protect mice against virus-induced changes in vascular permeability and inflammatory reactions (28, 30, 37, 38); thus, we were interested to know whether this anti-CLEC5A mAb can attenuate *P*. *aeruginosa*–induced lung inflammation and tissue damage. To address this question, mice were inoculated with a sublethal dose (LD_90_) of *P*. *aeruginosa* via an intratracheal route, followed by injection of the antibiotic ciprofloxacin alone or in conjunction with anti-CLEC5A mAb (Supplemental Figure 8); survival was monitored over 120 hours. Compared with isotype control (20% survival; Figure 6A), anti-CLEC5A mAb alone had a marginal effect against the sublethal dose of *P*. *aeruginosa* (30% survival; Figure 6A) and was far less effective than ciprofloxacin alone (70% survival; Figure 6A). However, the combination of anti-CLEC5A mAb and antibiotic almost completely prevented *P*. *aeruginosa–*induced lethality in mice (90% survival; Figure 6A). While cell infiltration to the lungs was still substantial at 5 days after inoculation in mice treated with ciprofloxacin alone (left, Figure 6B), the combination of anti-CLEC5A mAb and ciprofloxacin inhibited cell infiltration dramatically (Figure 6B, right). We further defined the populations of cells (39) inhibited by anti-CLEC5A mAb via in situ multiple-color staining, where fluorescence images (Figure 6C) were analyzed using MetaMorph software (Figure 6D). At day 5 after infection, massive infiltrations of CD11b^+^Siglec-F^–^F4/80^–^CD64^–^ neutrophils/DC (polymorphonuclear neutrophil/DC [PMN/DC]) and CD11b^+^Siglec-F^+^F4/80^+^CD64^–^ eosinophils (Eos), as well as increased numbers of CD64^+^CD11b^–^Siglec-F^+^F4/80^+^ AMФ and CD64^+^CD11b^+^Siglec-F^–^F4/80^+^ interstitial macrophages (IMФ) were noted in mice treated with ciprofloxacin (Figure 6, C and D). In contrast, all these populations were suppressed in mice treated with a combination of ciprofloxacin and anti-CLEC5A mAb (Figure 6E). We further confirmed this observation by isolating infiltrating cells for flow cytometry analysis. We found that addition of anti-CLEC5A mAb decreased the number of PMN, AMФ, Eos, and IMФ (Figure 6F and Supplemental Figure 9) compared with ciprofloxacin alone.

**Figure 7:**
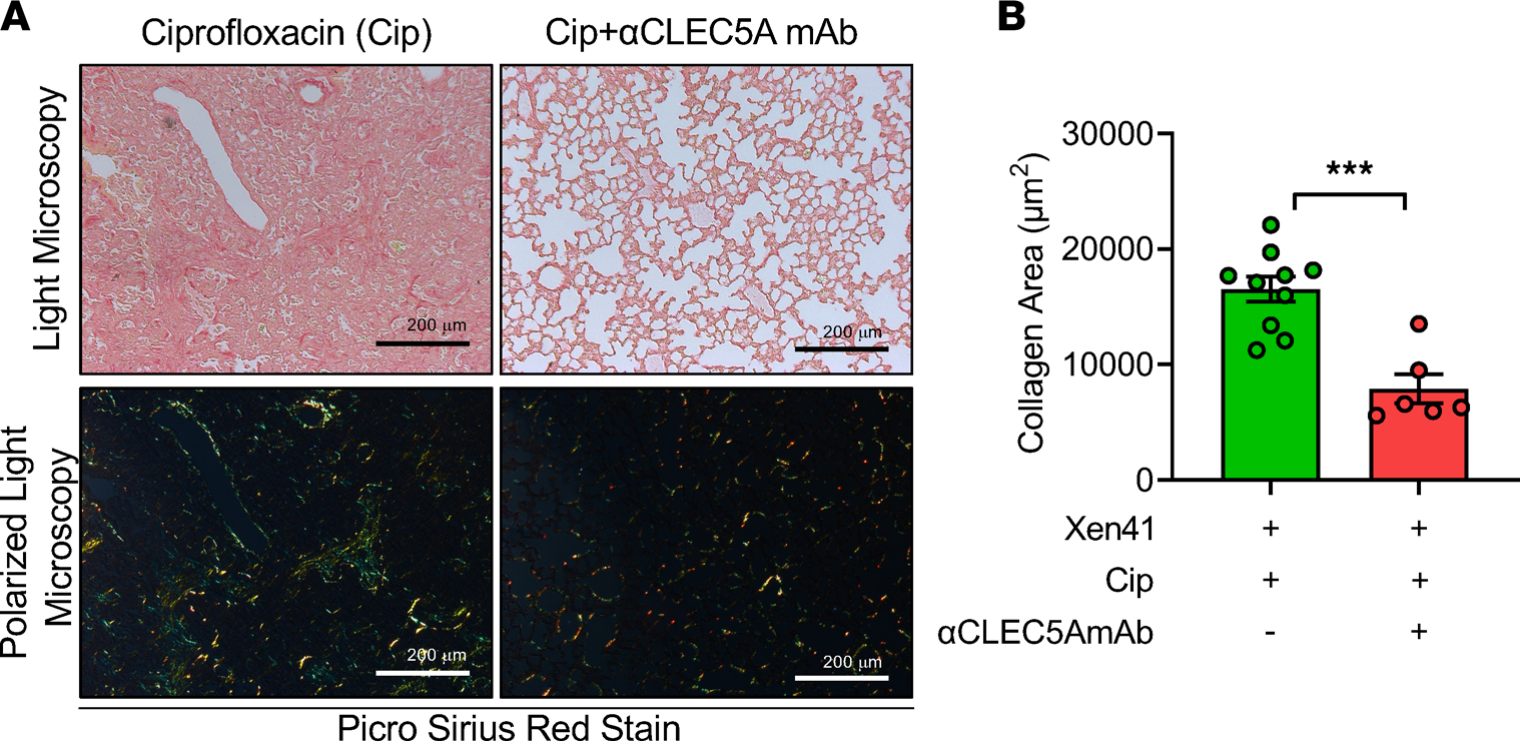
CLEC5A is a potential therapeutic target in *P*. *aeruginosa*–induced collagen deposition. WT mice were intratracheally injected with a sublethal dose (LD_90_) of *P*. *aeruginosa* Xen41 (1 × 10^6^ PFU) and injected with isotype control (mIgG1, 100 μg per mouse, i.v.), anti-mCLEC5A mAb (clone 4A12D5, 100 μg per mouse, i.v.), ciprofloxacin (Cip, 4 mg/kg, i.p.), or anti-mCLEC5A mAb combined with ciprofloxacin at 15 hours after infection. Ciprofloxacin was given every 12 hours. (**A**) Collagen deposition was observed by Picrosirius red staining, and the images were captured using light microscopy (upper panel) and polarized light microscopy (lower panel). Scale bar: 200 μm. (**B**) Collagen deposition was quantified from polarized light images using MetaMorph software and presented as area (μm^2^) of collagen. Data are mean ± SEM from Xen41 + Cip (*n* = 10) and Xen41 + Cip + anti-CLEC5A mAb (*n* = 6). Statistical analysis was calculated with unpaired and nonparametric Student’s *t* test with Mann-Whitney *U* test. ***P < 0.001.

To determine the extent of fibrosis following *P*. *aeruginosa* infection, lung sections were subjected to Picrosirius red staining and were observed under a microscope without or with polarized light (Figure 7A). For mice treated with ciprofloxacin alone, intense red coloring was observed, suggesting the deposition of collagen in lung tissue (Figure 7A, left upper panel). We also observe yellow-orange birefringence (type I collagen, thick fiber) and green birefringence (type III collagen, thin fiber) under polarized light (Figure 7A, left lower panel). In contrast, collagen deposition was inhibited dramatically in mice treated with ciprofloxacin and anti-CLEC5A mAb together (Figure 7A, right panels); the quantitation of collagen deposition in these 2 groups is shown in Figure 7B. All these observations suggest that blockade of CLEC5A is able to prevent lung inflammation and fibrosis after *P*. *aeruginosa* infection and is complementary to the bactericidal effect of ciprofloxacin.

## Discussion

It has been shown that neutrophils dominate the early immune response in pathogen-induced ALI. However, targeting of neutrophil infiltration to attenuate lung injury has not yet been tested. While *P*. *aeruginosa* secretes toxins that cause tissue damage, lung injury due to the innate immune response during acute *Pseudomonas* infection frequently manifests as ARDS. It is known that TLR4 and TLR5 are required for host defense and bacterial clearance following *P*. *aeruginosa* invasion, but the PRRs responsible for *P*. *aeruginosa*–induced lung damage and ARDS are still unclear. In this study, we demonstrate that *P*. *aeruginosa* activates CLEC5A to induce excessive NET formation, thereby causing pulmonary inflammation and collagen deposition. While TLR4 deficiency impairs phagocytosis and killing of *P*. *aeruginosa*, CLEC5A deficiency does not. These observations suggest that CLEC5A is a pathogenic factor during *P*. *aeruginosa* infection.

It is interesting to observe that *P*. *aeruginosa*–induced NET formation is impaired in both *Clec5a^–/–^* and *Tlr2^–/–^* neutrophils. It has been reported that TLR2 recognizes lipoprotein of *P*. *aeruginos*a (40); thus, neutrophils may be activated by the interactions of lipoprotein and LPS of *P*. *aeruginos*a with TLR2 and CLEC5A, respectively. This is consistent with our previous report that CLEC5A and TLR2 form a heterocomplex and are coactivated upon engagement with *L*. *monocytogenes* (31). Thus, TLR2 deficiency may impair the formation of CLEC5AS/TLR2 heterocomplexes, thereby attenuating *P*. *aeruginosa*–induced NET formation in *Tlr2^–/–^* neutrophils. This could explain why the anti-CLEC5A mAb only has a marginal effect on increasing survival rate in *P*. *aeruginosa*–infected mice (Figure 6A). It would be interesting to test whether simultaneous blockade of CLEC5A and TLR2 can further improve survival rate.

We were surprised to find that CLEC5A deficiency in mice was associated with reduced *P*. *aeruginosa* infiltration to the lung,but higher bacterial loads in blood, kidney, and spleen after intratracheal inoculation (Supplemental Figure 4). It is possible that reduced chemokine secretion in *Clec5a^–/–^* mice may result in less neutrophil infiltration to the lung (Figure 4), while the inability of *Clec5a^–/–^* neutrophils to trap *P*. *aeruginosa* in the lung and liver (Supplemental Figure 4C) might facilitate the spread of *P*. *aeruginosa* to the blood, spleen, and kidney.

CLEC5A has been shown to be upregulated in AMФ from cigarette smoke–exposed (CS-exposed) mice and is responsible for CS-induced pulmonary inflammation, macrophage activation, and lung remodeling. Thus, CLEC5A is regarded as a potentially novel therapeutic target for chronic obstructive pulmonary disease (COPD) (41). In this study, we also found that CLEC5A contributed to *P*. *aeruginosa*–induced pulmonary inflammatory reaction and tissue damage. Even though pulmonary EPCs do not express CLEC5A, proinflammatory cytokine production was attenuated in EPCs from *P*. *aeruginosa*–infected *Clec5a^–/–^* mice (Supplemental Figure 7). This observation suggests that myeloid cells partly depend on CLEC5A to induce cytokine release by EPCs. Moreover, blockade of CLEC5A by an antagonistic anti-CLEC5A mAb attenuates pulmonary inflammation and increases host survival during *P*. *aeruginosa* infection. Since cross-talk between AMФ and pulmonary EPCs is essential to maintain lung homeostasis (42, 43), these observations suggest that anti-CLEC5A mAb may block *P*. *aeruginosa*–induced AMФ activation, thereby reducing lung inflammation via attenuating proinflammatory cytokine release from AMФs and pulmonary EPCs.

Even though NET formation is beneficial to the host in controlling bacterial spreading, deficiency in protein arginine deiminase 4 (PAD4), which is critical for NET formation, does not affect bacteremia and survival in polymicrobial sepsis (44). Moreover, excessive NET formation correlates with poor outcomes in lung-related diseases, such as bacterial pneumonia infections, CF, and COPD (45). Moreover, antimicrobial peptide CRAMP, which is upregulated during inflammatory reactions, induces NET formation via CLEC5A (Figure 3G), suggesting that CLEC5A is a potentially novel receptor for CRAMP. Thus, activation of CLEC5A induces an autocrine feedback loop by increasing the release of CRAMP, which further stimulates CLEC5A to enhance *P*. *aeruginosa*–induced NET formation. It has been reported that LL-37 (the human ortholog of CRAMP) stimulates virulence factor production and enhances *P*. *aeruginosa* (PAO1 strain) resistance to fluoroquinolone (such as ciprofloxacin) and aminoglycoside antibiotics (46). Thus, blockade of CLEC5A may prevent antimicrobial peptide–induced resistance and enhance fluoroquinolone-mediated cytotoxic effects in vivo. This would explain our observation that a combination of anti-CLEC5A mAb and fluoroquinolone further reduced lung inflammation and increased survival, compared with antibiotic alone, in mice challenged with a lethal dose (LD_90_) of P. *aeruginosa* (Figure 6A).

Compared with WT neutrophils, we observed that *Clec5a^–/–^* neutrophils produced less NET formation in response to *P*. *aeruginosa* LPS (Figure 1A), while PMA-induced NET formation was similar both WT and *Clec5a^–/–^* neutrophils (Supplemental Figure 8B). *P*. *aeruginosa* activated CLEC5A to induce caspase-1–dependent NET formation in neutrophils (Figure 1, D, E, and G). Moreover, *P*. *aeruginosa* activated CLEC5A to induce proinflammatory cytokine release and caused caspase-1–dependent GSDMD cleavage in macrophages. Although caspase-1 is activated in both neutrophils and macrophages, GSDMD is only cleaved in macrophages — not neutrophils. These observations suggest that there are distinct CLEC5A-mediated signaling pathways in human neutrophils and macrophages.

Previously, we reported that CLEC5A is crucial for the host to clear *L. monocytogenes*, a Gram-positive intracellular bacterium causing severe liver inflammation (31). Therefore, it is evident that CLEC5A can mediate protective or pathogenic roles during microbial infections, depending on the distinct pathogenic mechanisms of each microbe (47). While activation of CLEC5A facilitates the killing of intracellular bacteria in macrophages (31), we have shown here that activation of CLEC5A by *P*. *aeruginosa* induced severe neutrophil infiltration and NET formation in pulmonary tissues. In our recent study, we found that NETs contribute to detrimental changes in systemic vascular permeability after dengue virus infections (38). Similarly, *P*. *aeruginosa* caused extensive NET formation and cellular infiltration via CLEC5A; blockade of CLEC5A reduced these effects without attenuating TLR-mediated cytotoxicity, thereby attenuating lung damage and increasing host survival after infection with *P*. *aeruginosa*. Previously, NET formation was reported to confine *P*. *aeruginosa* biofilm formation to the cornea and prevent spreading to brain (12); this observation further supports the notion that NETs may be beneficial or detrimental during microbial infections depending on the distinct microenvironment of each organ.

The development of alternatives to antibiotic treatments is a major goal, aiming to reduce off-target effects and address the challenge of multidrug resistance. Meyer et al. proposed that attenuation of the host inflammatory response may be of equal or greater importance than antibacterial chemotherapy in preventing progressive lung destruction in CF (48). We have found that antibody-mediated blockade of CLEC5A not only reduces lung inflammation, but also protects the host from *P*. *aeruginosa*–induced lung inflammation. The beneficial effects of administering antibodies at 15 hours after inoculation of mice with *P*. *aeruginosa* further suggest that anti-CLEC5A mAb is a promising nonantibiotic therapeutic agent to alleviate lung inflammation and damage during acute *P*. *aeruginosa* infection. Moreover, the combination of anti-CLEC5A mAb with antibiotics not only reduced lung inflammation in acute pneumonia, but also prevented lung fibrosis and increased host survival rate. Thus, blockade of CLEC5A is a promising strategy to prevent progressive lung destruction in patients with CF and those with COPD in the future.

## Methods

### Reagents and antibodies.

LPS isolated from *E*. *coli* O111:B4 (catalog L2630) and *K. pneumoniae* 15380 (catalog L4268), as well as ciprofloxacin, were purchased from MilliporeSigma. LPS from *Pseudomonas aeruginosa* PAO1 was isolated by the Tri-Reagent method as described previously (49), and the purity of the various LPS preparations is shown in Supplemental Table 1. Pam3CSK4 (tlrl-pms) was purchased from InvivoGen. Granulocyte macrophage–CSF (GM-CSF) was from R&D Systems. Murine CRAMP (mCRAMP) was purchased from AnaSpec (catalog AS-61305). Caspase-1 inhibitor Z-WEHD-FMK (catalog FMK002) was purchased from R&D Systems, and caspase-11 inhibitor wedelolactone (catalog W4016) was purchased from MilliporeSigma. Primary antibodies for IHC staining were sourced as follows: anti-F4/80 Ab (ab74383, Abcam), anti-MPO Ab (AF3667, R&D Systems), anti-CD11b Ab (ab133357, Abcam), anti–Cit-H3 Ab (NB100-57135, NOVUS), anti–Siglec-F Ab (PA5-11675, Invitrogen), and anti-CD64 Ab (MA5-29704, Invitrogen). Secondary antibodies were obtained from the following suppliers: peroxidase AffiniPure donkey anti–rabbit IgG (H+L) (711-035-152, Jackson ImmunoResearch), donkey anti–goat IgG-HRP (sc-2020, Santa Cruz Biotechnology Inc.). Antibodies for flow cytometry include: APC-conjugated anti-CD45 Ab (559864, BD Pharmingen), BV510-conjugated anti-Ly6G Ab (127633, BioLegend), PE/Cy7-conjugated anti-CD45 Ab (103114, BioLegend), PerCP/Cy5.5-conjugated anti-CD64 Ab (139308, BioLegend), BV421-conjugated anti–Siglec-F Ab (155509, BioLegend), APC/Cy7-conjugated anti-IA/IE Ab (107628, BioLegend), APC-conjugated anti-CLEC5A Ab ( FAB1639R, R&D), APC-conjugated anti–IL-6 Ab (504508, BioLegend), BV510-conjugated anti–TNF-α Ab(506339, BioLegend), PE-conjugated anti–pro–IL-1β Ab (12-7114-82, eBioscience), PerCP/Cy5.5-conjugated anti-CD31 Ab (102440, BioLegend), BV421-conjugated anti-CD326 Ab (118210, BioLegend), BUV805-conjugated anti-CD45 Ab (752415, BD Pharmingen), BV650-conjugated anti-Ly6G Ab (127641, BioLegend), BV480-conjugated anti-CD11c Ab (565627, BD Pharmingen), BUV395-conjugated anti-CD11b Ab (565976, BD Pharmingen), BV605-conjugated anti-CD64 Ab (139323, BioLegend), PE-Cy5–conjugated anti-CD24 Ab (15-0242-82, Thermo Fisher Scientific), BUV496-conjugated anti-IA/IE Ab (750281, BD Pharmingen), and PE-conjugated anti-RANTES Ab (149104, BioLegend).

### Bacterial strains and culture.

*Pseudomonas aeruginosa* Xen41 (bioluminescent *P*. *aeruginosa* strain PAO1) was purchased from PerkinElmer, and strain PAO1 was a gift from Richard Cumming (Harvard University, Cambridge, Massachusetts, USA). Before inoculation, Xen41 was refreshed in BHI broth for 1.5 hours at 37°C, according to the PerkinElmer manufacturer manual. The concentration of Xen41 was measured by the determination of absorbance at OD 600 nm (0.3 OD = 1 × 10^8^ CFU/mL).

### Isolation of murine primary cells.

Mouse BM cells were isolated from femurs and tibias, and the RBCs were lysed before further culture. For isolation of BMDMs, BM cells were cultured in RPMI containing 10% (v/v) FBS with 10 ng/mL M-CSF at 37°C for 7 days. Isolation of BM-derived neutrophils was described previously (38).

### Induction of NETs.

Neutrophils (2 × 10^5^ cells in 100 μL) were seeded on poly-L–lysine–coated coverslips; they were then incubated with PAO1 (MOI = 3 or 10) or LPS (10 μg/mL) at 37°C for 1 hour. Alternatively, neutrophils were incubated with CRAMP (50 μg/mL) at 37°C for 3 hours. Samples were fixed and stained with Hoechst 33342, anti-MPO Ab, and anti-histone Ab. Image capture and analysis were as described previously (38). In brief, for each sample, at least 5 images were randomly captured at 40× magnification using a Leica confocal microscope with a white light laser system (TCS-SP8-MP-SMD). NET formation was quantified from histone images using MetaMorph software and presented as area (μm^2^).

### Stimulation of BMDMs.

BMDMs (1 × 10^5^) were incubated with PAO1 or LPS from different bacteria (100 ng/mL) at 37°C for 1 hour; they were then incubated with RPMI containing gentamicin (Thermo Fisher Scientific, 15750060, 10 μg/mL) for another 30 minutes to kill bacteria. Cells were washed and resuspended in fresh RPMI containing 10% (v/v) FBS at 37°C for 24 hours, and the supernatant was harvested to measure cytokine levels by ELISA. For mRNA detection, BMDMs (1 × 10^6^) were incubated with PAO1 (MOI = 3 or 10) or LPS (100 ng/mL) for at 37°C for 1 hour, followed by addition of gentamicin (10 μg/mL) and incubated for another 30 minutes. Cells were lysed by TRIzol reagent (700 μL) to harvest RNA at 4 hours after incubation.

### IHC staining.

Samples were fixed in 10% paraformaldehyde for 24 hours and embedded in paraffin. The paraffin-embedded specimens were deparaffined by xylene and rehydrated using an ethanol gradient (100%, 95%, 85%, 75%, 50%, and 30%). For antigen retrieval, sections were boiled in citric acid (0.01M, pH 6.0) for 10 minutes twice; they were then cooled to room temperature. Endogenous peroxidase was quenched by Peroxidase Suppressor (Pierce Peroxidase Detection kit, Thermo Fisher Scientific, catalog 36000) for 30 minutes at room temperature. After blocking with Universal Blocker Blocking Buffer in TBS (Pierce Peroxidase Detection kit) for 30 minutes, specimens were incubated with anti-MPO Ab (1:100) and anti–Cit-H3 (1:100) at 4°C overnight. Multiple fluorescent staining was performed using the Opal 7-Color Manual IHC Kit (PerkinElmer, NEL811001KT) according to the vendor’s instruction. Images were captured using a Leica confocal microscope with a white light laser system (TCS-SP8-MP-SMD) and analyzed using Leica Application Suite X software.

### Inoculation of P. aeruginosa in mice.

*P. aeruginosa* Xen41 was refreshed in BHI medium at 37°C for 1.5 hours, and bacterial titer was measured at OD 600 (0.3 OD = 1 × 10^8^ CFU/mL). Mice were anesthetized with Avertin (240 μg/per g) and intratracheally challenged with *P. aeruginosa* Xen41 in 10 μL of saline. For anti-CLEC5A mAb treatment, WT mice were intratracheally challenged with a sublethal dose of Xen41 (1 × 10^6^ PFU in 10 μL of saline); this was followed by i.v. injection with anti-mCLEC5A mAb (clone 4A12D5, 100 μg in 100 μL) or i.p. injection with ciprofloxacin (4 mg/kg) or simultaneous injection at 15 hours after infection. Ciprofloxacin was reinjected every 12 hours until 4 days after infection. Survival was monitored until 120 hours after infection.

### Quantification of mRNAs and proteins in lung and bronchoalveolar lavage fluid.

Lung tissues were lysed with by TRIzol to harvest RNA for real-time PCR. The sequences of primers are shown in Supplemental Table 2. Data for each gene were normalized to GAPDH and presented as fold change (compared with mock). The conditions for real-time PCR were 95°C for 5 minutes, followed by 40 cycles of 15 seconds at 95°C, 30 seconds at 58°C, and 30 seconds at 72°C. To harvest bronchoalveolar lavage fluid (BALF), mice were anesthetized by urethane (0.6 g/mL, 50 μL per mouse), a 14G catheter on 1 mL syringe was inserted into the trachea, and 3 mL saline was applied (0.5 mL per installation); samples were centrifuged at 250*g* for 5 minutes at 4°C to remove cells. Cytokines and chemokines were quantified using ELISA kits (R&D Systems and MyBioSource, respectively) according to the suppliers’ instructions. Total protein concentrations in BALF samples were measured using DC protein assay (Bio-Rad, 5000116) according to the vendor’s instructions.

### Preparation of mouse lung cell suspensions for flow cytometry.

Mice were intratracheally challenged with *P. aeruginosa* (LD_50_, 3 × 10^5^ PFU) for 15 hours, and they were then i.p. injected with 150 μg of monensin (in 100 μL of 20% ethanol) at 12 hours before harvest. Mice were anesthetized with urethane (1.2 g/kg), and lungs were perfused with 10 mL of 0.5 mM EDTA in PBS before the sample collection. The preparation of lung cell suspension was described in the previous study (50). In brief, 1 mL of 0.5 mM EDTA in PBS was used to lavage the lung; then, 1 mL of digestion medium I (cold RPMI containing 4 U/mL elastase [LS002292, Worthington], 1 U/mL dispase [D4693, MilliporeSigma], and 200 μg/mL DNase I [D5025, MilliporeSigma]) was injected intratracheally. The trachea was tied tightly with silk thread and was then incubated in 3 mL of digestion medium I at 37°C for 45 minutes. The trachea was then removed, and the lungs were cut into small pieces (1 mm or smaller) in 3 mL of cold RPMI, followed by incubation in 3 mL of digestion medium II (cold RPMI containing 25 μg/mL Liberase [5401119001, Roche]) at 37°C for 30 minutes. The media were collected and centrifuged at 500*g* for 5 minutes at 4°C, and RBC lysis buffer was used to remove RBCs. Cells in suspension were blocked with 2.4G2 (10 μg/mL, BD Pharmingen, 553142) according to the vendor’s instruction and stained with Fixable Viability Stain 510 (BD Pharmingen, 564406), PE/Cy7-conjugated anti-CD45 Ab, PerCP/Cy5.5-conjugated anti-CD64 Ab, BV421-conjugated anti–Siglec-F Ab, APC/Cy7-conjugated anti-IA/IE Ab, APC-conjugated anti-CLEC5A Ab, FITC-conjugated anti–IL-6 Ab, PE-conjugated anti–TNF-α Ab, and PE-conjugated anti–pro–IL-1β Ab for AMФ gating strategy. For alveolar EPC staining, blocked cells were stained with Fixable Viability Stain 510, PE/Cy7-conjugated anti-CD45 Ab, PerCP/Cy5.5-conjugated anti-CD31 Ab, BV421-conjugated anti-CD326 Ab, and APC-conjugated anti-CLEC5A Ab. Samples were subjected to flow cytometry (BD FACSVerse) and analyzed with software FlowJo version 10. The gating strategies for AMФ, lung EPCs, and neutrophils are illustrated in Supplemental Figures 3–5.

### Western blotting.

For neutrophil analyses, cells (5 × 10^5^/mL) were stimulated with live PAO1 or UV-killed PAO1 (MOI = 3 and 10, respectively) for 1 hour at 37°C. For inflammasome activation, neutrophils were primed with LPS from *E*. *coli* (0.5 μg/mL, MilliporeSigma, L2630) for 3 hours at 37°C and were then activated with ATP (3 mM) or nigericin (10 μM) for 45 minutes at 37°C. The neutrophil lysates were harvested with 150 μL of RIPA containing proteinase inhibitor (cOmplete EDTA-free Protease inhibitor Cocktail, Roche), phosphatase inhibitor (PhosSTOP, Roche), and 2 mM phenylmethanesulfonyl fluoride (PMSF).

For BMDM analyses, cells (1 × 10^6^/mL) were incubated with live PAO1 or UV-killed PAO1 (MOI = 3 and 10, respectively) for 1 hour at 37°C; then, residual bacteria were removed, and cells were washed twice with RPMI containing gentamicin (10 μg/mL). They were further cultured in fresh RPMI containing 10% FBS and gentamicin (10 μg/mL) for 15 hours at 37°C. For inflammasome priming and activation, cells were pretreated with Pam3CSK4 (1 μg/mL) for 4 h or LPS (1 μg/mL, MilliporeSigma, L2630) for 3 hours at 37°C and were then transfected with LPS (2.5 μg/mL, MilliporeSigma, L2630) using 0.25% FuGENE reagent (Promega, E2311) for 15 hours. Cell lysates were harvested with 100 μL of RIPA containing proteinase inhibitor (cOmplete EDTA-free Protease inhibitor Cocktail, Roche), phosphatase inhibitor (PhosSTOP, Roche), and 2 mM PMSF. Cell lysates (15–25 μg) were fractioned on 12% SDS-PAGE under reducing conditions before transfer onto 0.45 μm PVDF membrane (Pall Corporation). After blocking with 5% skimmed milk in TBST for 1 hours at room temperature, membranes were hybridized with anti-GSDMD Ab (1:1,000, ab209845, Abcam), anti–caspase-11 Ab (1:1,000, ab180673, Abcam), anti–caspase-3 p12 Ab (1:1,000, ab179517, Abcam), anti–caspase-1 (p20) Ab (1:1,000, AG-20B-0042, AdipoGen), or anti-GAPDH Ab (1:500, MAB374, MilliporeSigma) overnight at 4°C. This was followed by incubation with peroxidase-conjugated donkey anti–rabbit IgG Ab (1:10,000, 711-035-152, Jackson ImmunoResearch) or peroxidase-conjugated donkey anti–mouse IgG Ab (1:10,000, 715-035-150, Jackson ImmunoResearch) for 1 hour at room temperature. SuperSignal West Femto Maximum Sensitivity Substrate kit (Thermo Fisher Scientific) was used for detection.

### Vascular permeability determination.

Mice were challenged with LD_50_ of Xen41 and i.v. injected with Evans Blue (0.1% in PBS, 100 μL) at 14 hours after infection. Lungs were perfused with 20 mL of saline before harvest, and the Evans Blue was extracted using 2 mL of formamide at 55°C for 5 hours. Evans Blue content was calculated and presented as μg/per lung.

### Flow cytometry.

Cells from BALF or lung cell suspensions were washed with FACS buffer (0.1% FBS and 0.1% NaN_3_ in PBS) and blocked with purified rat anti–mouse CD16/CD32 (553141, BD Pharmingen) according to the vendor’s instruction. Neutrophils in BALF were stained with APC-conjugated anti-CD45 Ab and BV510-conjugated anti-Ly6G Ab; after washing, cells were resuspended in FACS buffer, and the CD45^+^Ly6G^+^ neutrophils were detected by flow cytometry. Lung cell suspensions were stained with BUV805-conjugated anti-CD45 Ab, BV650-conjugated anti-Ly6G Ab, BV480-conjugated anti-CD11c Ab, BUV395-conjugated anti-CD11b Ab, BV605-conjugated anti-CD64 Ab, PE-Cy5-conjugated anti-CD24 Ab, and BUV496-conjugated anti-IA/IE Ab, and cell populations were determined by flow cytometry (BD FACSVerse).

### μCT analysis.

Mice were challenged with Xen41 (LD50; 3 × 10^5^ PFU), and at 15 hours after infection, lung images were captured by μCT using a SkyScan1276 instrument (at 55 kV, 200 μA) and analyzed using CTAn software (Version 1.0.12; Bruker). Three-dimensional models were generated using cone-beam reconstruction from 536 images per sample (image size = 828 × 828 pixels).

### Collagen deposition.

Lung sections were deparaffined and rehydrated before being stained with Picrosirius red Stain Kit (ab150681, Abcam); images were captured using a light microscope with polarized light (Nikon). Quantification of collagen was performed by MetaMorph, and the level of collagen deposition was presented as area (μm^2^) of collagen.

### Bacterial phagocytosis and killing assays.

Neutrophils (1 × 10^5^/50 μL) were incubated with PAO1 (MOI = 0.1 or 1) in RPMI containing 10% FBS for 1 hour at 37°C. Cells were centrifugated at 500*g* for 5 minutes at 4°C to remove unengulfed bacteria and were then washed twice with 1 mL of saline. Cell pellets were resuspended in 100 μL of RPMI containing 5% FBS and incubated for 1 hour at 37°C. Cells were then washed twice with saline, pelleted by centrifugation (500*g* for 5 minutes at room temperature), and lysed by vortexing for 5 minutes in 100 μL of sterile H_2_O. Culture supernatants and cell lysates were incubated on LB agar plates at 37°C for 20–24 hours, and bacterial colonies were counted. Phagocytosis (%) was quantified using the formula: (Input bacteria number – unengulfed bacteria number)/input bacteria number × 100%. Bacterial killing (%) was quantified using the formula: (engulfed bacteria number – unkilled bacteria number)/engulfed bacterial number × 100%.

### Determination of bacterial burden in vivo.

WT and *Clec5a^–/–^* mice were intratracheally inoculated with LD_50_ Xen41 and sacrificed at the desired time point. Organs were weighed and homogenized using MagNA Lyser Green Beads and MagNA Lyser (Roche). Bacterial load in homogenates was measured following incubation on LB agar plates at 37°C for 20–24 hours.

### Animals.

All mice were maintained in the specific pathogen–free animal facility in Academia Sinica SPF animal facility and the Laboratory Animal Center of National Defense Medical Center. C57BL/6J WT mice were purchased from the National Laboratory Animal Center, while the *Clec5a^–/–^* and *Tlr2^–/–^* mice were generated as described previously (38). *Tlr4^–/–^* mice were a gift from Tsung-Hsien Chuang (National Health Research Institutes). *Clec5a^–/–^* mice and littermates were cohoused before experiments.

### Statistics.

All data were analyzed using GraphPad Prism software (Version 8.0) and presented as dot plots with bar charts. For 2-group comparisons, a 2-tailed unpaired *t* test was used. For multiple-group comparisons, parametric data were analyzed by 2-way ANOVA with the Bonferroni post hoc test; nonparametric data were analyzed by Kruskal–Wallis analysis with Dunn’s post hoc test. The survival rate was assessed using Kaplan–Meier analysis. *P* < 0.05 was considered significant.

### Study approval.

Mice used in this study were 8–12 weeks old, and experiments were approved by the IACUC (Academia Sinica; protocol no. 15-109-872).

## Author contributions

PSS designed, performed experiments, and wrote manuscript. SPY and YCP performed experiments and data analysis. CHC contributed discussion and supplied materials. SLH designed experiments, interpreted data, and wrote the manuscript.

## Supplementary Material

Supplemental data

## Version 1 09/01/2022

In-Press Preview

## Version 2 09/22/2022

Electronic publication

## Version 3 11/20/2022

Corrected publication date in PDF
