# Supplementary material for: CLEC5A is critical in Pseudomonas aeruginosa–induced NET formation and acute lung injury
Source: JCI Insight. 2022 Sep 22;7(18):e156613. doi: 10.1172/jci.insight.156613 (PMC9676025; doi:10.1172/jci.insight.156613)
Supplement: Supplemental data [file jciinsight-7-156613-s008.pdf]

Figure S1.

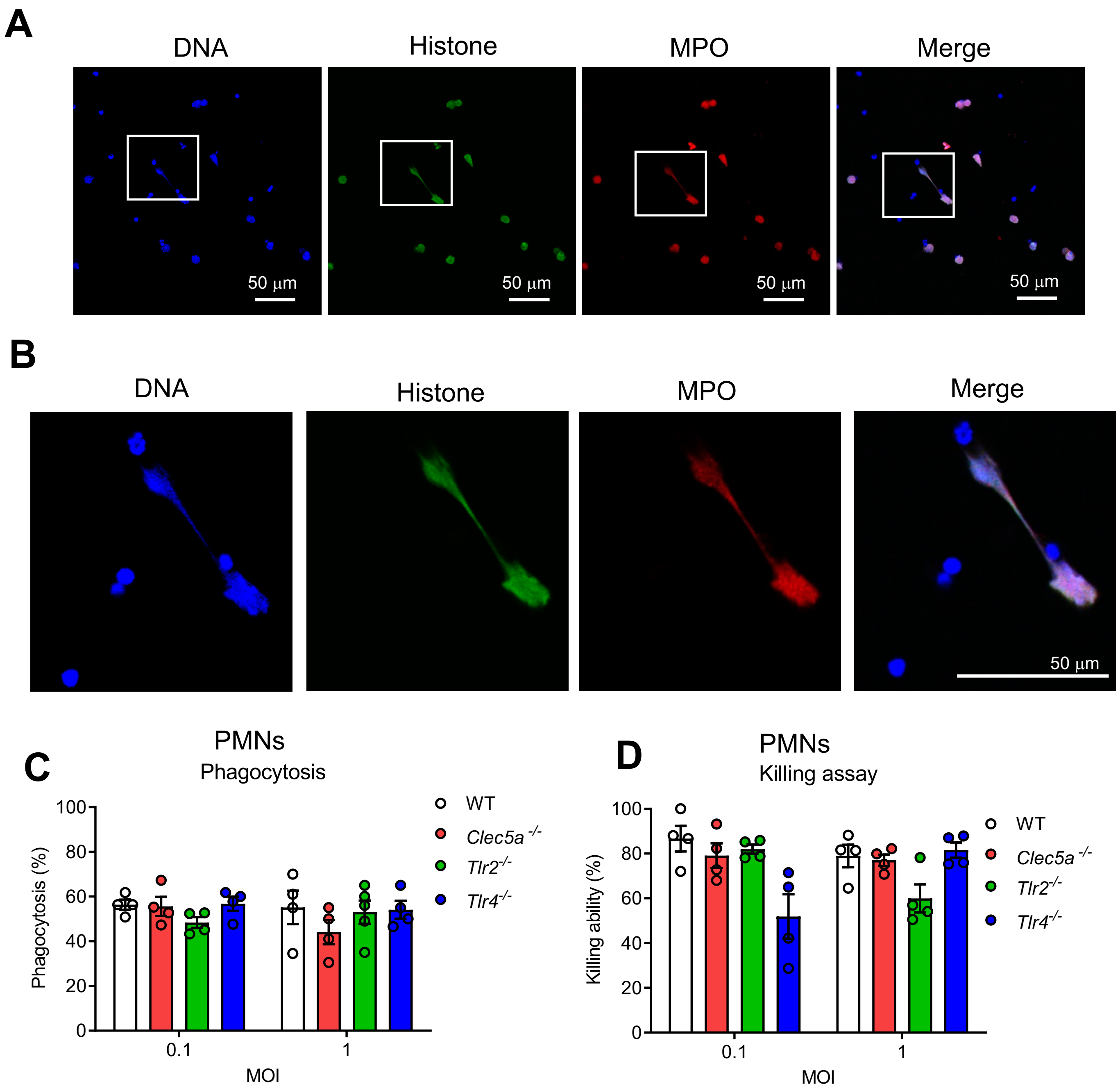
A
DNA
Histone
MPO
Merge
50 μm
B
DNA
Histone
MPO
Merge
50 μm
C
PMNs
Phagocytosis
Phagocytosis (%)
0
20
40
60
80
100
0.1
1
MOI
WT
Clec5a -/-
Tlr2-/-
Tlr4-/-
D
PMNs
Killing assay
Killing ability (%)
0
20
40
60
80
100
0.1
1
MOI
WT
Clec5a -/-
Tlr2-/-
Tlr4-/-

Figure S2.

Short exposure of Caspase1 in Fig. 2C

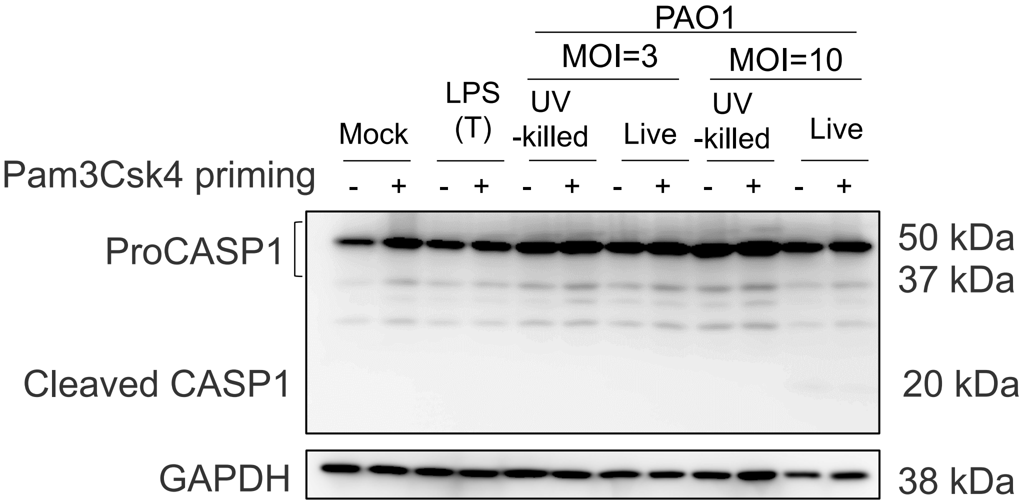

Figure S3.

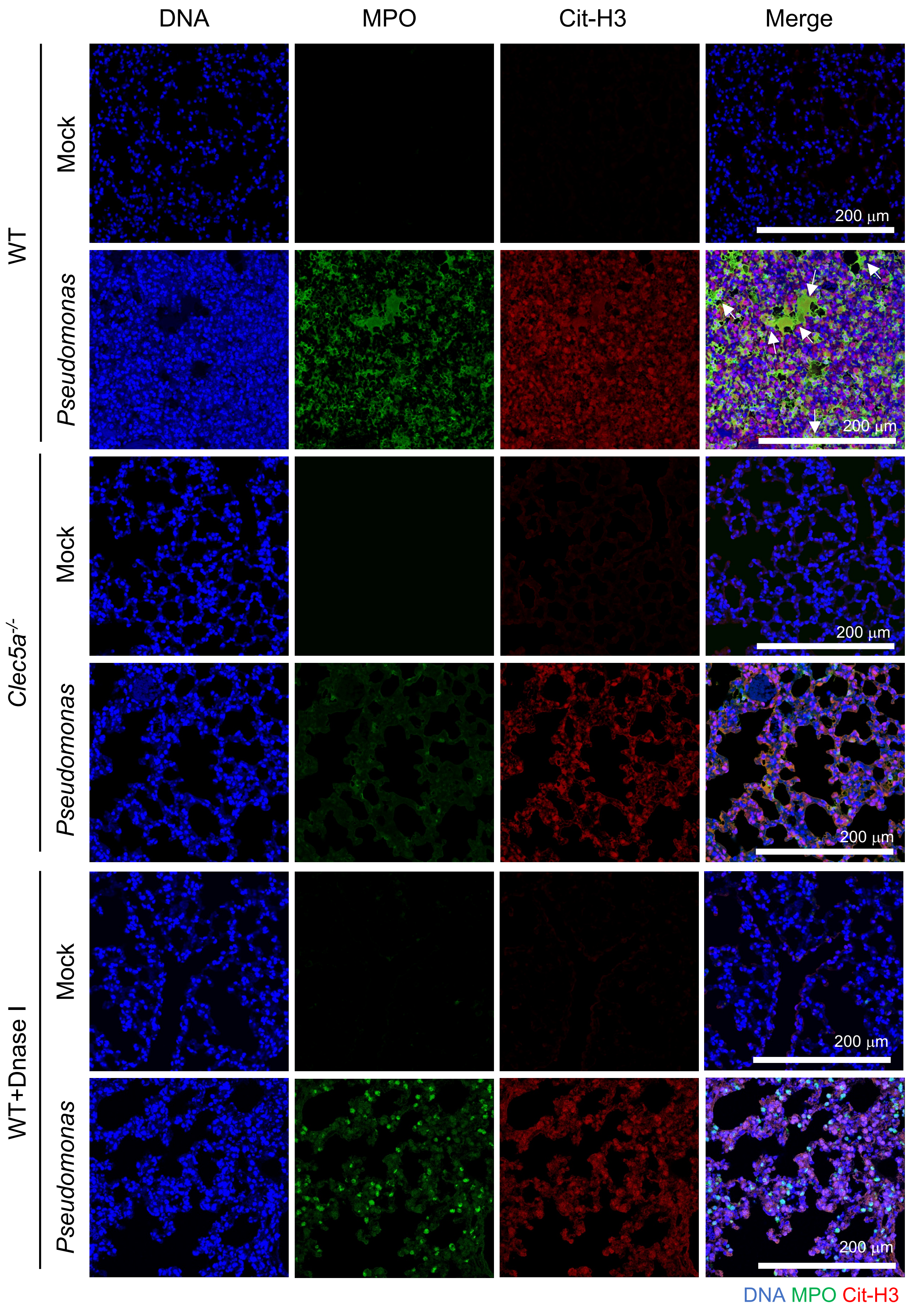

DNA
MPO
Cit-H3
Merge
WT
Mock
Pseudomonas
Clec5a-/-
Mock
Pseudomonas
WT+Dnase I
Mock
Pseudomonas
200 μm
200 μm
200 μm
200 μm
200 μm
200 μm
DNA MPO Cit-H3

Figure S4.

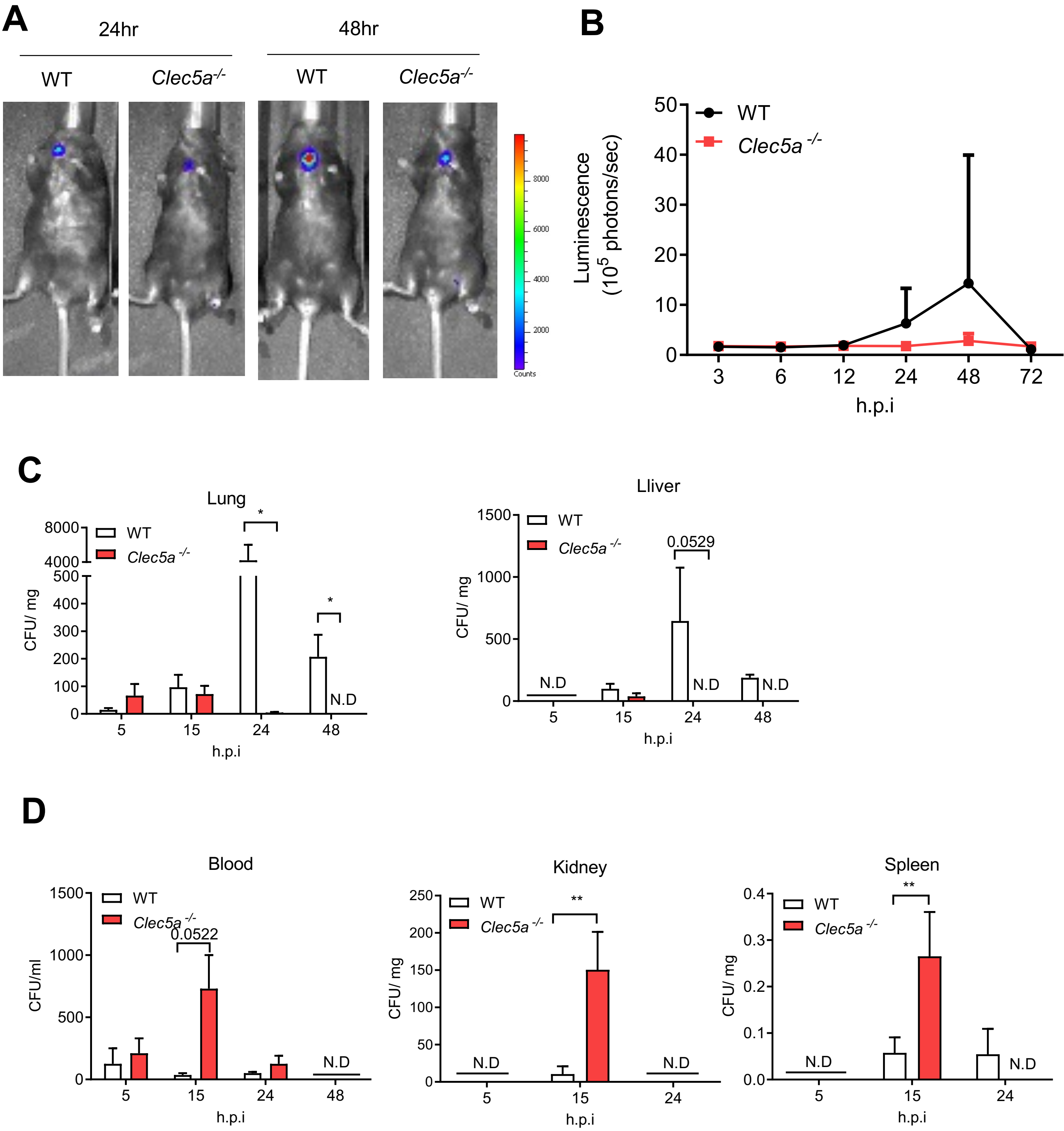
A
24hr
48hr
WT
Clec5a-/-
WT
Clec5a-/-
8000
6000
4000
2000
Counts
B
WT
Clec5a -/-
Luminescence (10^5 photons/sec)
50
40
30
20
10
0
3
6
12
24
48
72
h.p.i
C
Lung
WT
Clec5a -/-
CFU/ mg
8000
4000
500
400
300
200
100
0
*
*
N.D
5
15
24
48
h.p.i
Lliver
WT
Clec5a -/-
CFU/ mg
1500
1000
500
0
0.0529
N.D
N.D
N.D
N.D
5
15
24
48
h.p.i
D
Blood
WT
Clec5a -/-
CFU/ml
1500
1000
500
0
0.0522
N.D
5
15
24
48
h.p.i
Kidney
WT
Clec5a -/-
CFU/ mg
250
200
150
100
50
0
**
N.D
N.D
5
15
24
h.p.i
Spleen
WT
Clec5a -/-
CFU/ mg
0.4
0.3
0.2
0.1
0.0
**
N.D
N.D
5
15
24
h.p.i

Figure S5.

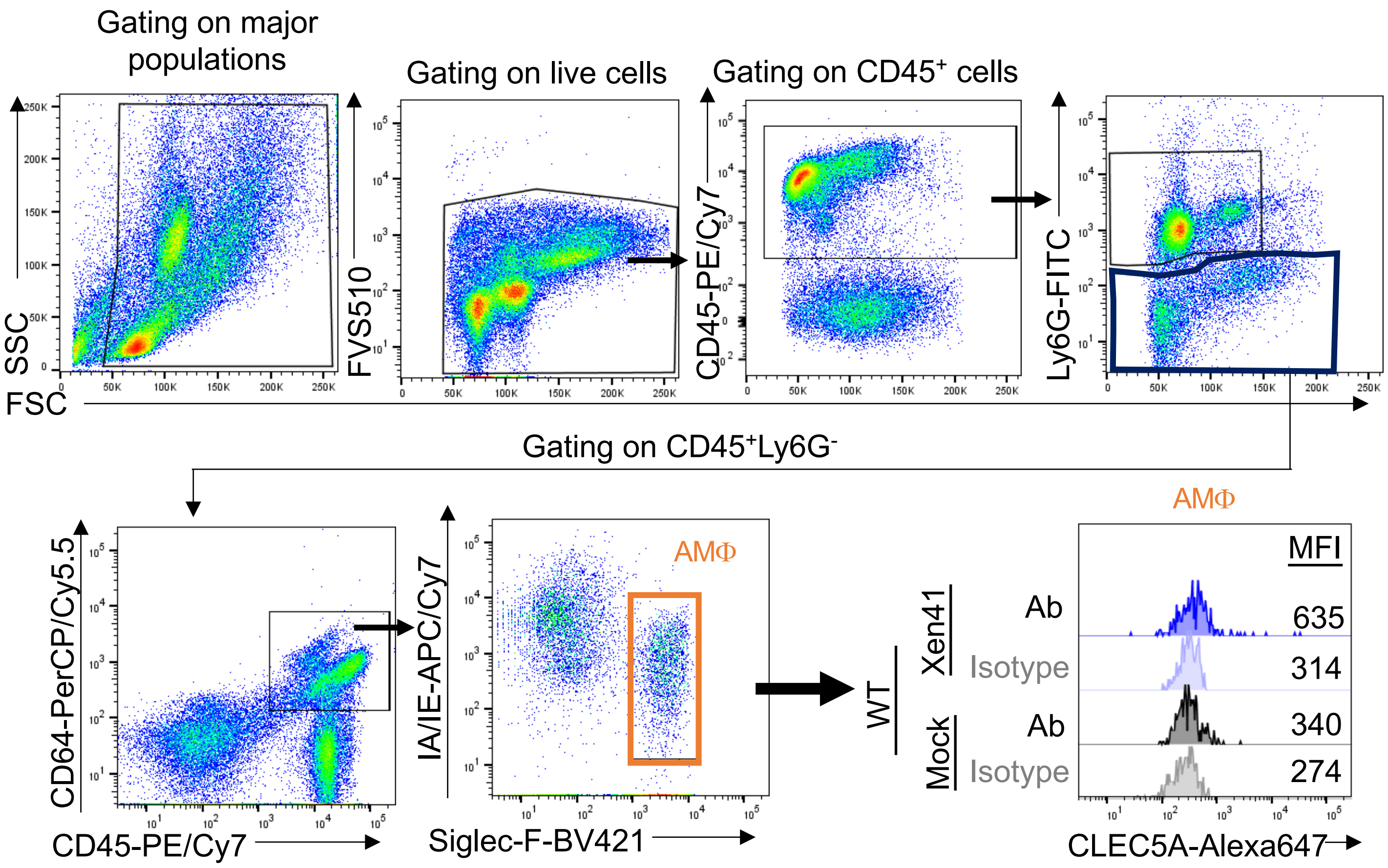

Gating on major populations
Gating on live cells
Gating on CD45⁺ cells
SSC
FSC
FVS510
CD45-PE/Cy7
Ly6G-FITC
Gating on CD45⁺Ly6G⁻
CD64-PerCP/Cy5.5
CD45-PE/Cy7
IA/IE-APC/Cy7
Siglec-F-BV421
AMΦ
AMΦ
MFI
WT
Xen41
Mock
Ab
Isotype
Ab
Isotype
635
314
340
274
CLEC5A-Alexa647

Figure S6.

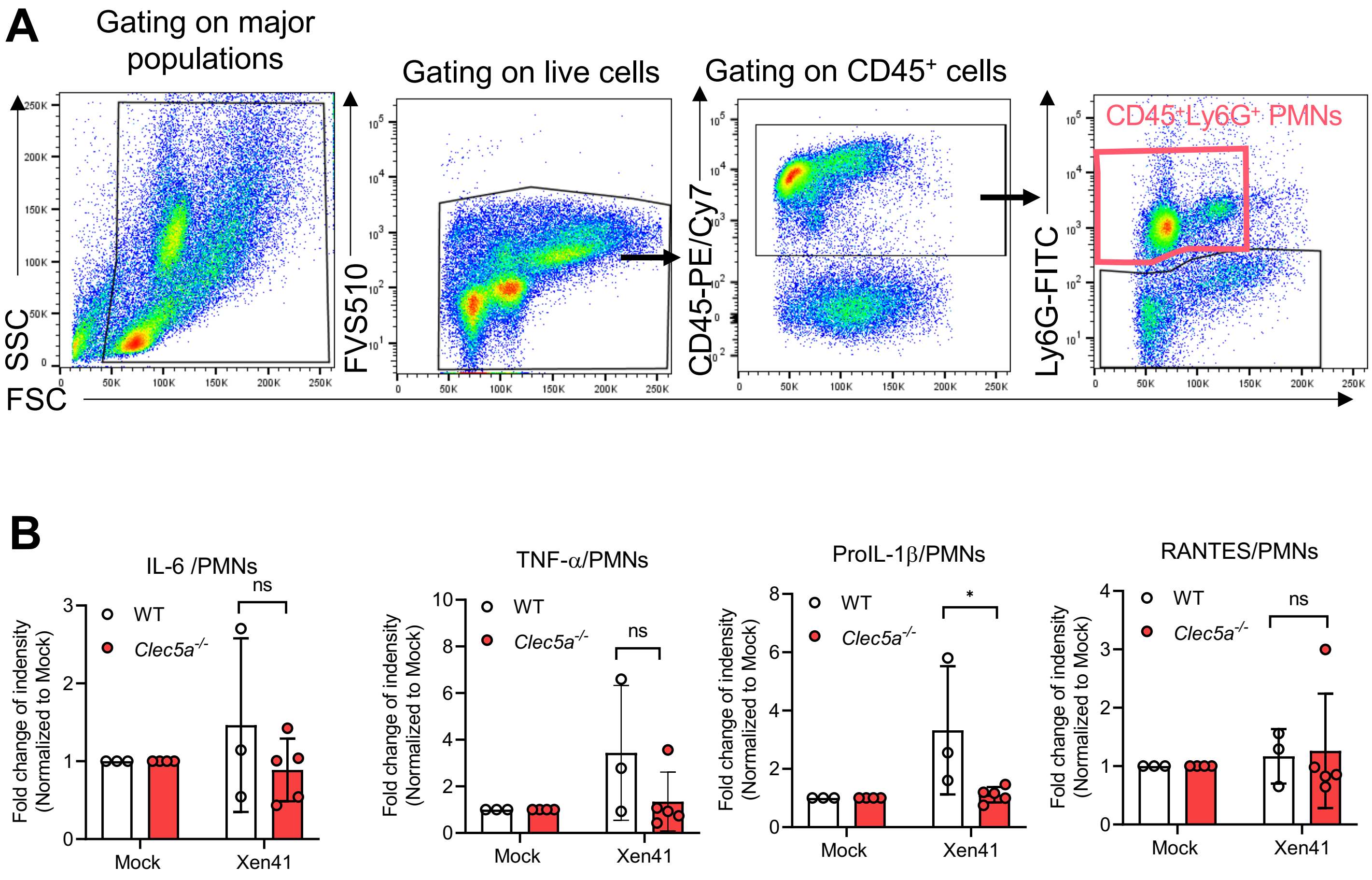
A
Gating on major populations
Gating on live cells
Gating on CD45+ cells
SSC
FSC
FVS510
CD45-PE/Cy7
Ly6G-FITC
CD45+Ly6G+ PMNs
B
IL-6 /PMNs
TNF-α/PMNs
ProIL-1β/PMNs
RANTES/PMNs
WT
Clec5a-/-
ns
*
Fold change of indensity (Normalized to Mock)
Mock
Xen41

Figure S7.

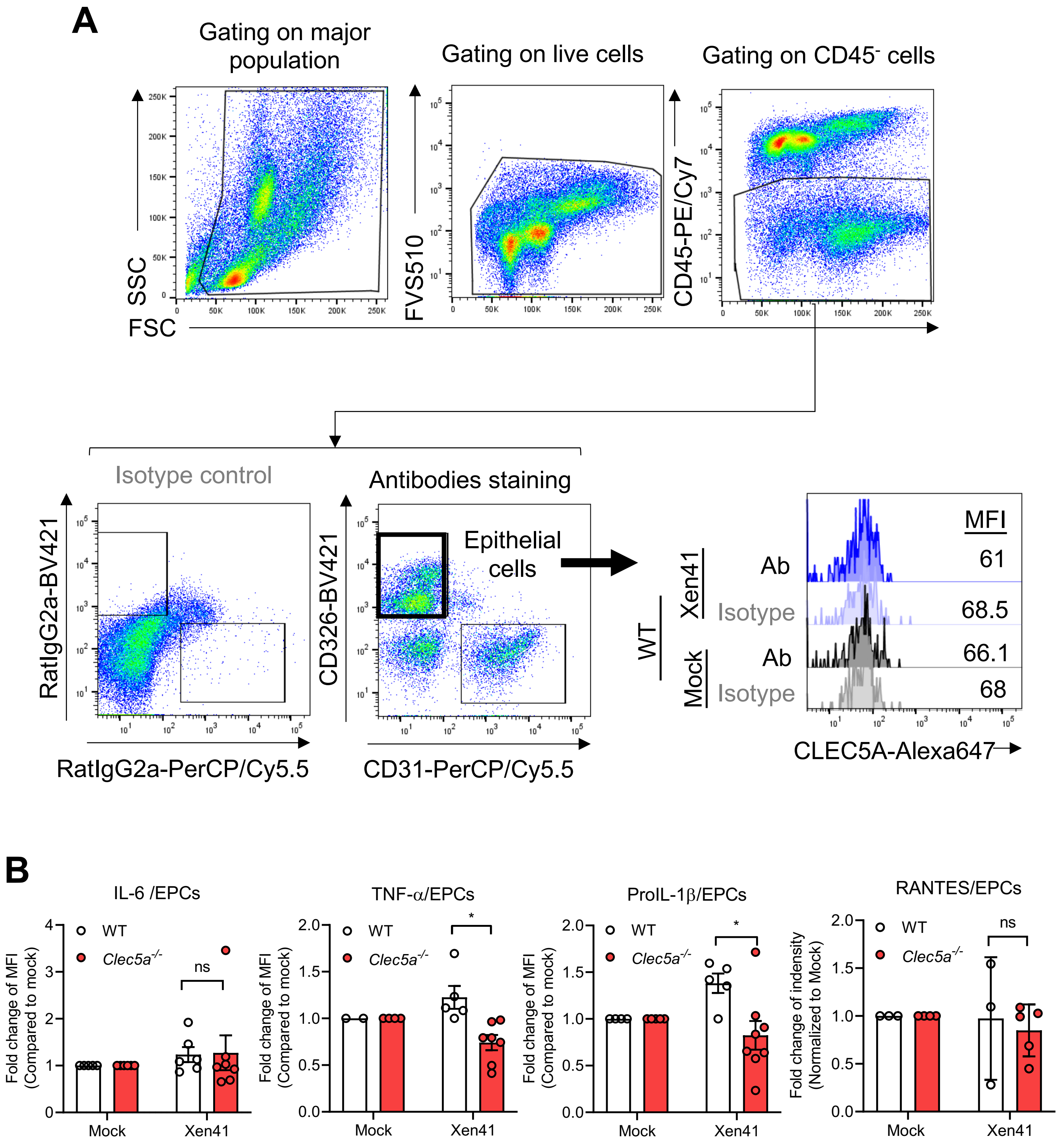
A
Gating on major population
Gating on live cells
Gating on CD45- cells
SSC
FSC
FVS510
CD45-PE/Cy7
Isotype control
Antibodies staining
RatIgG2a-BV421
RatIgG2a-PerCP/Cy5.5
CD326-BV421
CD31-PerCP/Cy5.5
Epithelial cells
MFI
WT
Xen41
Mock
Ab
Isotype
61
68.5
66.1
68
CLEC5A-Alexa647
B
IL-6 /EPCs
TNF-α/EPCs
ProIL-1β/EPCs
RANTES/EPCs
WT
Clec5a-/-
ns
*
Fold change of MFI (Compared to mock)
Fold change of indensity (Normalized to Mock)
Mock
Xen41

Figure S8.

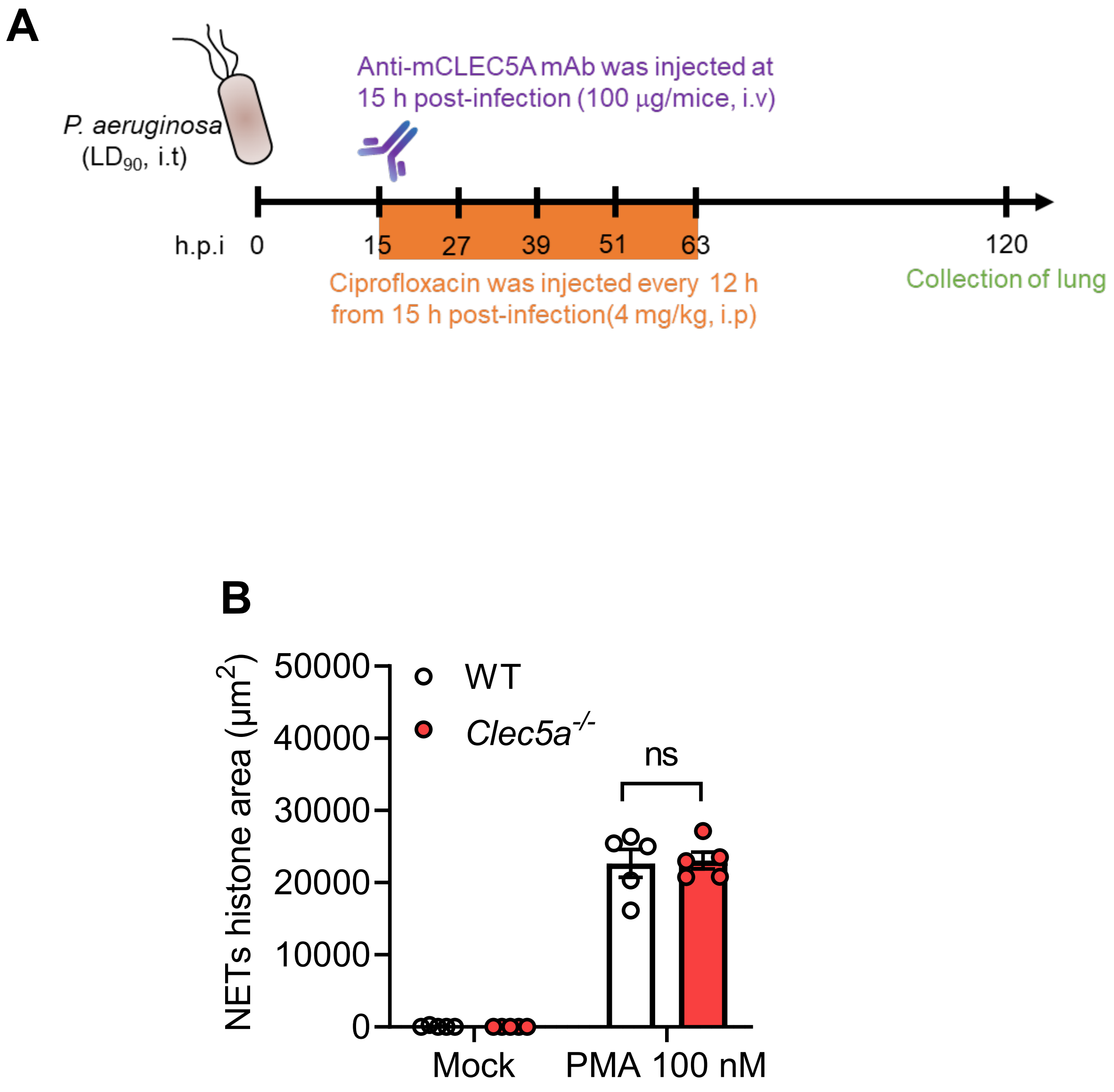
A
P. aeruginosa
(LD90, i.t)
Anti-mCLEC5A mAb was injected at
15 h post-infection (100 μg/mice, i.v)
h.p.i 0 15 27 39 51 63 120
Ciprofloxacin was injected every 12 h
from 15 h post-infection(4 mg/kg, i.p)
Collection of lung
B
NETs histone area (μm2)
50000
40000
30000
20000
10000
0
WT
Clec5a-/-
ns
Mock
PMA 100 nM

Figure S9.

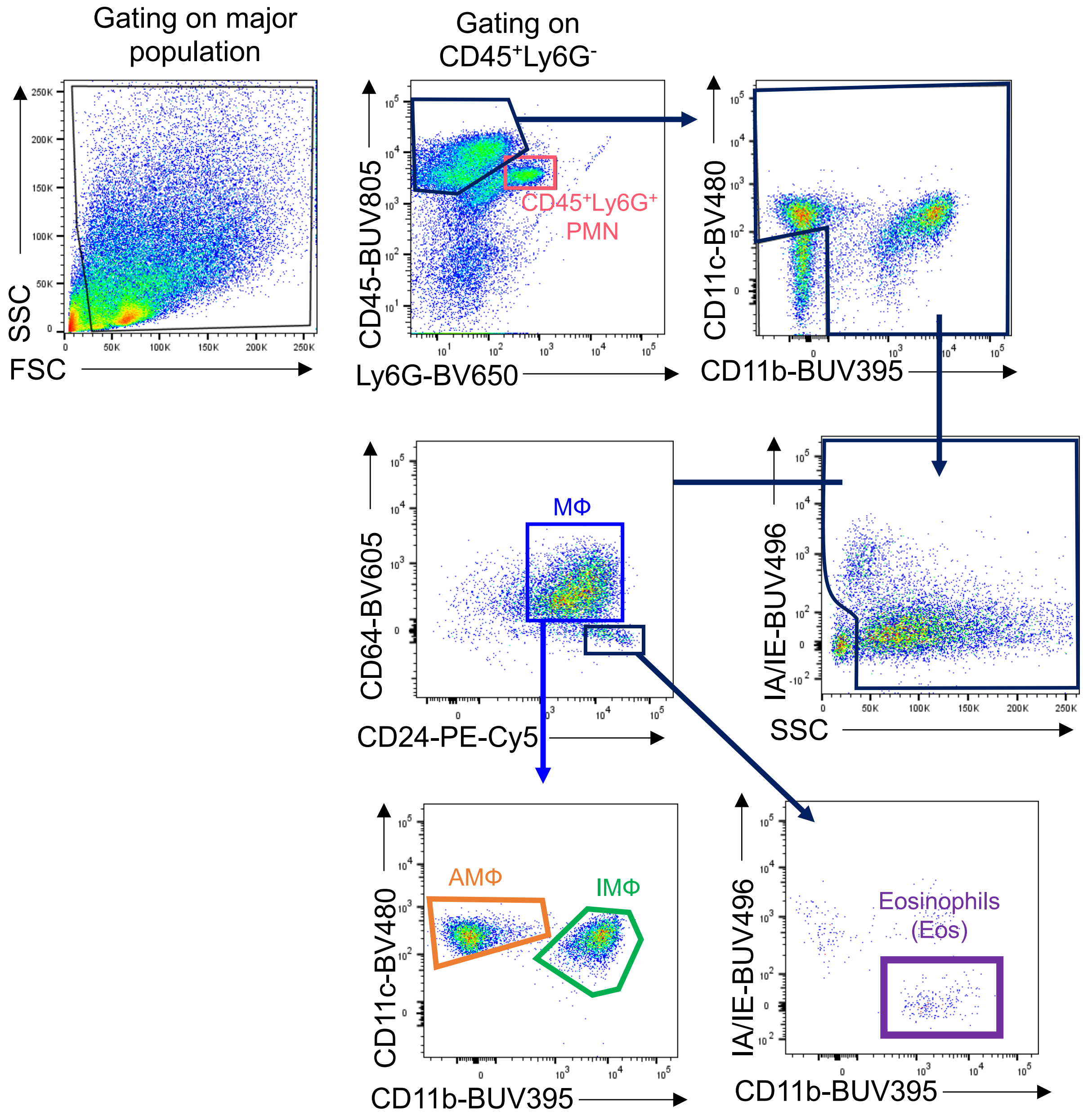
Gating on major population
SSC
FSC
Gating on CD45⁺Ly6G⁻
CD45-BUV805
Ly6G-BV650
CD45⁺Ly6G⁺ PMN
CD11c-BV480
CD11b-BUV395
CD64-BV605
CD24-PE-Cy5
MΦ
IA/IE-BUV496
SSC
CD11c-BV480
CD11b-BUV395
AMΦ
IMΦ
IA/IE-BUV496
CD11b-BUV395
Eosinophils (Eos)

Suppl. Table 1. Protein concentration and percentage in LPS.

	LPS (10 μg/ml)		
	E. coli	*K. pneumoniae*	*P. aeruginosa* (PAO1)
Protein concentration (μg/ml)	0.65172	1.14952	0.07532
Protein percentage (%)	6.5172	11.4952	0.7532

Suppl. Table 2. Sequences of qPCR primers

Target	Forward sequence (5' to 3')	Reverse sequence (5' to 3')
mouse *Tnfa*	GCCTCTTCTCATTCCTGCTTG	CTGATGAGAGGGAGGCCATT
mouse *Il6*	GAGGATACCACTCCCAACAGACC	AAGTGCATCATCGTTGTTCATAC
mouse *Il1b*	GGAGAACCAAGCAACGACAAAATA	TGGGGAACTCTGCAGACTCAAAC
mouse *Cxcl1*	CAATGAGCTGCGCTGTCAGTG	CTTGGGGACACCTTTTAGCATC
mouse *Cxcl2*	CCAAGGGTTGACTTCAAGAAC	AGCGAGGCACATCAGGTACG
mouse *Cramp*	GCCGCTGATTCTTTTGACAT	GCCAAGGCAGGCCTACTACT
mouse *Gapdh*	GGAGAAACCTGCCAAGTATG	TGGGAGTTGCTGTTGAAG
